# ﻿Life history and biogeography of the enigmatic mantid *Nilomantisfloweri* (Mantodea, Nanomantidae)

**DOI:** 10.3897/zookeys.1173.107204

**Published:** 2023-08-04

**Authors:** Zohreh Mirzaee, Saber Sadeghi, Francesco Ballarin, Thomas Schmitt, Marianna Simões, Martin Wiemers

**Affiliations:** 1 Senckenberg German Entomological Institute, Eberswalder Str. 90, 15374 Müncheberg, Germany Senckenberg German Entomological Institute Müncheberg Germany; 2 Biology Department, Faculty of Sciences, Shiraz University, Shiraz, Iran Shiraz University Shiraz Iran; 3 Entomology and Biogeography, Institute of Biochemistry and Biology, Faculty of Science, University of Potsdam, D-14476 Potsdam, Germany Tokyo Metropolitan University Tokyo Japan; 4 Systematic Zoology Laboratory, Department of Biological Sciences, Tokyo Metropolitan University, 1-1 Minami-Osawa, Hachioji-shi, 192-0397, Tokyo, Japan Tokyo Metropolitan University Tokyo Germany; 5 Senckenberg Research Institute and Natural History Museum, Frankfurt, Germany Senckenberg Research Institute and Natural History Museum Frankfurt Germany

**Keywords:** Climatic niche, desert species, faunistics, Nilomantini, western Asia

## Abstract

The biology and distribution patterns of the Sahelian mantid species *Nilomantisfloweri* are still insufficiently known. For the first time, records are confirmed of this species from Iran and the distribution map of its native range is updated. Records are compiled from the Sahel zone of North Africa, the Arabian Peninsula, and Iran. Detailed information on its biology, oothecal characteristics, male genitalia variation, and intraspecific molecular diversity in the mitochondrial gene cytochrome c oxidase are provided, and ecological niche modelling was used to gain insight into the overall species distribution and understand its climatic niche limits. Genetic analysis revealed only one haplotype shared between Iran and Oman. The Iranian populations likely represent two distinct clusters, both more related to the diverse Oman haplotypes than to each other. Based on new data, *N.floweri* appears to be mostly associated with coastal areas in southwestern Asia, with the vast majority of records found along the Red Sea, Persian Gulf, and Oman Gulf coasts. This distribution contrasts markedly with *N.floweri* records in the Sahel, where most collections have been reported in the transitional zone between the southern Sahara and arid thorn savannah, far off the coast. This study contributes to a comprehensive understanding of this still enigmatic mantid species.

## ﻿Introduction

**Figure 1.**
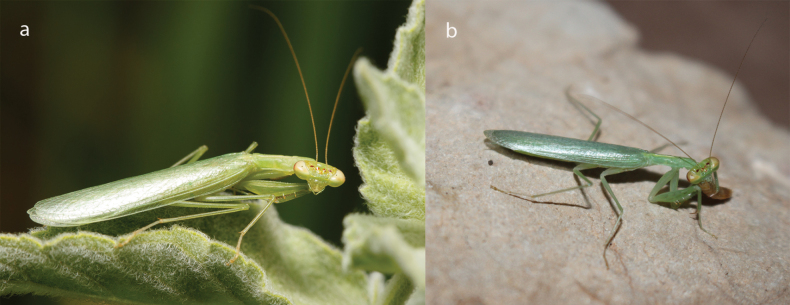
*Nilomantisfloweri* Werner, 1907: **a** female habitus **b** male habitus.

Mantids are a fascinating group of animals and have attracted a lot of attention from scientists and laymen with the consequence of good knowledge about the biology, distribution, and taxonomy of many species (Ehrmann 2002). Nevertheless, despite the popularity of this insect group, some species still remain enigmatic due to their difficulty to be found and rarity in the field. One of these examples is *Nilomantisfloweri* Werner, 1907 (Mantodea: Nanomantidae). This mantid is a small, slender species (total body length 15–21 mm) with fully developed wings in both sexes (Fig. 1). Its known distribution extends throughout the southern Sahara–Sahel zone of Africa (Mauritania to Eritrea) and the Arabian Peninsula (all major countries; Table 1). So far, the only known mention beyond this region refers to Iran (Kaltenbach 1982; Ehrmann 2002) but does not include any proof. In addition to distributional uncertainty, few studies exist that address in detail the taxonomy of this species (Giglio-Tos 1915; Beier 1930; Uvarov 1939; Roy and Leston 1975; Kaltenbach 1982; Brannoch and Svenson 2016).

**Table 1.** Known records and the reported number of specimens (NOS) of *Nilomantisfloweri* per country with years of collection, * the collection from 1901 goes back to before the establishment of this genus and species by Werner in 1907. On a trip to Iran, Zarudny collected one male and one female of this species from Makran in Sistan and Baluchistan Province and deposited them at the Zoological Institute of the Russian Academy of Sciences, Saint Petersburg. Evgeny Shcherbakov (Lomonosov Moscow State University, Russia) recently identified them as *Nilomantisfloweri*.

Country	Year	NOS	Reference
Chad	1958	3	Roy and Leston 1975
Eritrea	1907, 1931	3	NRM, Roy and Leston 1975
Iran	1901*–2021	35	This study
Niger	1897, 1958	4	Roy and Leston 1975
Oman	1976, 2000, 2016, 2017, 2020, 2022	45	Roy and Leston 1975, GBIF, iNaturalist, Kaltenbach 1982
Qatar	2020	1	iNaturalist
Saudi Arabia	1930–1932, 1934, 1936, 1938, 1940, 1945, 1948, 1956, 1962, 1975–1980, 2019	43	Roy and Leston 1975, GBIF, iNaturalist, Kaltenbach 1982
Sudan	1906, 1928, 1931, 1957	4	Roy and Leston 1975, LMZ
United Arab Emirates	2014, 2015, 2019–2022	18	iNaturalist
Yemen	1987, 1988, 1945, 1956, 1996, 1998, 2000	50	SMNK, Roy and Leston 1975

External morphology, male genitalia, and geographic distribution have traditionally been used to describe and classify mantid species. Nevertheless, high intraspecific variability in male genital characteristics makes it difficult to separate some closely related species. In addition, intraspecific morphological variability is still unknown or poorly documented for numerous species. In *N.floweri* for instance, a high variation in the shape of the phalloid apophysis was first pointed out by Roy and Leston (1975). However, because there is a shortage of studies, its systematic relevance remains largely unexplored. In addition, the lack of knowledge of the life history of the species, or application of techniques like phylogeographic molecular analysis, has hindered the understating of mechanisms underlying such variation. As a consequence, the intraspecific structure of *N.floweri* remains mostly unknown.

Moreover, limited information is available regarding the distributional boundaries of the species. Scattered records are reported in the literature, but lack exact coordinates, and based on its known distribution, sampling gaps are suspected, especially within the African range of the species. In this context, ecological niche models (ENMs) may help to better understand the species’ distribution and shed light on potential sampling gaps.

To fill the knowledge gaps observed in *N.floweri*, we provide new and updated information on the life history, intraspecific differentiation, and biogeography by providing new data on its distribution, life history traits, ootheca characteristics, morphological variation of male genitalia, and phylogeography. In doing so, we analysed newly collected material from Iran, confirming its presence in this country, and explored the intraspecific morphological and genetic diversity among southwestern Asian populations to improve the general knowledge about this species. In addition, we used ENM to gain insight into its distribution via its climatic niche.

## ﻿Materials and methods

### ﻿Field collection and laboratory rearing

**Figure 2.**
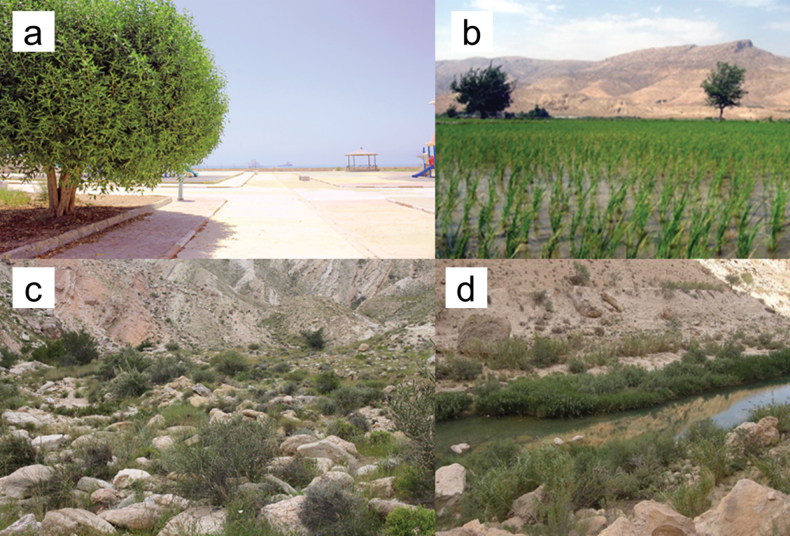
Examples of habitats where *N.floweri* was found in Iran: **a** urban park in Bandar-e Lengeh, Hormozgan Province **b** paddy field in Firuzabad, Fars Province **c** scree in Jam **d** rocky river bank in Kangan, Bushehr Province.

Nine specimens were collected at night by searching the leaves of *Conocarpus* trees that were planted in urban parks near the sea (Iran: Bandar Bushehr, Bandar-e Lengeh, Kangan, Chahbahar). Six specimens were observed in paddy fields, urban parks, or in mountains with natural springs during day excursions (Firuzabad, Meymand, Jam, Tonbak) (Figs 2, 8). Fresh specimens were collected by hand during day surveys or captured using an insect net or light traps during night collections. All samples were preserved in 96% ethanol for morphological and molecular analysis. Oothecae were collected searching in the vegetation during both night and day time.

Female specimens were collected alive in the field and kept in glass jars (15 × 15 × 10 cm) in order to increase the number of available oothecae. These samples were preferentially selected based on their large abdomens assuming that they have previously mated in their natural habitat. Laid oothecae were kept at room temperature (25–27 °C) in separate glass jars (15 × 15 × 10 cm). To simulate their natural dry environment, the relative air humidity (RH) was kept at 50–55% by misting the room on a regular basis. RH was measured using an HTC2 digital terrarium hygrometer (Dongguan, China).

Newly hatched nymphs were housed in separate glass jars (6 × 6 × 4 cm) aiming to obtain information on their life cycle and the number of instars of the species. A woody stick was placed into each jar to help the mantid hang on while moulting. Every three days, they were fed with one to two fruit flies (*Drosophilamelanogaster* Meigen, 1830) or small ants (*Trichomyrmex* spp.). Living mealworm larvae (*Tenebriomolitor* Linnaeus, 1758) were fed to later instars twice a week.

### ﻿Morphological analyses

Adult specimens were examined under a Leica M205 C stereomicroscope and identified to species level in accordance with Brannoch and Svenson (2016), Roy and Leston (1975), and Werner (1907). Brannoch et al. (2017) were followed for the preparation of male genitalia. We dissected the ultimate segments of the male abdomen under the microscope, separated genitalia from terminalia, and macerated the genitalia in 10% KOH solution for 12 hours. Subsequently, we washed the sample for 24 hours in distilled water, followed by 70% ethanol, and finally, glycerine to remove the remaining ethanol. The genitalia were then photographed and placed in a vial with glycerine drops for further study. The photographs were taken using a system that included the Stone Master Stack Unit, an Olympus OM-D E-M1 Mark II camera, and Zeiss Luminar lenses (40 mm). Olympus Capture and Stone Master v. 3.8 software were among the programs used. Helicon Focus 7 (https://www.heliconsoft.com/) was used for photo stacking, and ImageJ 1.53t (https://imagej.nih.gov/ij/index.html) was used to add the scale bars.

The material used in this study is preserved in the following collections: **ESPC** (personal collection Evgeny Shcherbakov, Ramenskoye, Russia), **SDEI** (Senckenberg German Entomological Institute, Müncheberg, Germany), **ZIN** (Zoological Institute of the Russian Academy of Sciences, Saint-Petersburg, Russia), **ZMPC** (personal collection Zohreh Mirzaee, Kangan, Iran), **ZMCBSU** (Zoological Museum of Shiraz University, Iran), and **VGPC** (personal collection Valeriy Govorov, Prague, Czech Republic).

### ﻿Distribution records

Distributional data of *N.floweri* were assembled through fieldwork, museum collections, publications, and online biodiversity databases. In total, 34 records were obtained from various districts of southern Iran over a five-year survey period (2017–2021); 47 occurrences were obtained from museum collections, including the  State Museum of Natural History Karlsruhe, Germany (**SMNK**), the Swedish Museum of Natural History, Sweden (**NRM**), and the Insect collections Inventory of the Lund University Biological Museum, Sweden (**MZLU**) (all museum specimens checked by mantid specialists, i.e., Ehrmann, Roy, Kaltenbach); 35 records were obtained from the Global Biodiversity Information Facility (GBIF; https://doi.org/10.15468/dl.twk7e4), 18 from iNaturalist, from which only specimens containing barcode information, or photographs which allowed a proper species identification (confirmed by ZM), were included; 30 additional records were obtained from Roy and Leston (1975) and Kaltenbach (1982).

In total, we obtained 164 occurrence records (Suppl. material 1: table S1). They were rarefied through spatial thinning using the R package “spThin”’ (Aiello-Lammens et al. 2015) using a 10-km distance, considering the spatial resolution of variables (~ 9.2 km at the equator) (Kramer-Schadt et al. 2013), to avoid problems derived from spatial autocorrelation. This resulted in a final count of 73 records used to calibrate and create the final models. Dubious records were corrected (e.g., for the reversed latitude and longitude fields), or removed following the protocol in Cobos et al. (2018). All records were used to generate a distribution map in QGIS v. 3.22 (https://qgis.org/en/site/). Google Earth v. 9.174.0.2 (https://earth.google.com/web/) was used to georeference specimens without coordinates based on the information present on the corresponding labels.

### ﻿Environmental data

Environmental data were obtained from WorldClim v. 2.0 (http://worldclim.org/version2; Fick and Hijmans 2017) at 2.5 arc-minute spatial resolution. WorldClim is based on interpolations of weather station data over the period 1950–2000. Of the 19 available bioclimatic variables, the variables 8, 9, 18, and 19 (namely, the mean temperature of wettest quarter, mean temperature of driest quarter, precipitation of warmest quarter, and precipitation of coldest quarter) were excluded from the analysis due to spatial artifacts between adjacent grid cells in those variables (Escobar et al. 2014; Campbell et al. 2015). To avoid overfitting and inflation of model accuracy with overly dimensional environmental space and collinearity among variables, we performed a jackknife analysis to evaluate the relevance of variables, the Pearson’s correlation coefficient (r) analysis, where variables with a correlation of > 0.8 were excluded, followed by the variance inflation factor (VIF) (USDM R package) (Naimi et al. 2014).

To test the best set of environmental variables, and create the final ecological niche models (ENMs), three environmental sets were used to calibrate our models, based on fieldwork observations by ZM of potentially biologically meaningful variables related to the *N.floweri* distribution: ‘set 1’, including the maximum temperature of the warmest month (Bio5), minimum temperature of the coldest month (Bio6), annual precipitation (Bio12), precipitation of driest quarter (bio17); ‘set 2’, containing annual mean temperature (Bio1), the maximum temperature of the warmest month (Bio5), annual precipitation (Bio12), precipitation of driest month (Bio14); and ‘set 3’, containing maximum temperature of the warmest month (Bio5), minimum temperature of the coldest month (Bio6), annual precipitation (Bio12), precipitation of driest month (Bio14).

### ﻿Climate suitability modelling

The maximum entropy algorithm in Maxent v. 3.3.3e (Phillips et al. 2006) was used to estimate the climatic suitability for *N.floweri*. A calibration area (Barve et al. 2011) of 50 km buffer around each distributional record was used to represent areas that this species could potentially exploit. The estimation of a 50-km buffer is based on the observation of this species in its natural habitat, especially for males, which have efficient wings and fly to find females in order to mate. Seeking to adjust model complexity, we first calibrated our models by creating 84 candidate models, with parameter combinations including four regularisation multipliers (0.1, 0.5, 1.0, and 1.5), seven feature classes that represent combinations of linear (L), quadratic (Q), and product (P), and three sets of environmental variables. Selected model parameterisations were the ones that showed statistical significance (partial Receiver Operating Characteristic (ROC) < P-value; with *E* = 5%, 500 iterations, and 50% of data for bootstrapping; Peterson et al. 2008), omission rates below a predetermined error rate (*E* = 5%; Anderson et al. 2003), and the lowest value of the Akaike information criterion corrected for small sample size (AICc; Warren and Seifert 2011).

The selected predictors were then used to create final model projections for the current climatic scenario, with ten replicates by bootstrap, logistic outputs, enabling MaxEnt to perform clamping and extrapolation. Binary maps were created from MaxEnt continuous median models using the ten-percentile training presence value as a criterion for viewing and comparing the extent of areas assessed as the possible distribution of *N.floweri*. The three occurrence data sets were randomly split into training (75% of the data) to calibrate the models, and test (25% of the data) to evaluate the models. All modelling processes were performed using the ‘*kuenm*’ package (Cobos et al. 2019) in R v. 3.6.2 (R Core Team 2022). Quantum GIS v. 3.22.9 (QGIS Development Team 2022) was used to generate and visualise maps. The modelling protocol followed that of Simões et al. (2020).

To evaluate the risk of strict extrapolation, we used the kuenm_mmop function (Cobos et al. 2019). This function determines mobility-oriented parity (MOP) layers (Owens et al. 2013) by contrasting the environmental values of the calibration area with those of one or more other regions or situations where ecological niche models were applied. The resulting layers generated by this function assist in identifying areas where there is a high risk of extrapolation, as well as various levels of similarity between the projection regions or scenarios and the calibration area.

### ﻿Phylogeographic analyses

Mesocoxal muscle tissue was excised from specimens preserved in 96% ethanol. Total genomic DNA was extracted using the E.N.Z.A Tissue DNA Kit protocol for animal tissue. Amplification of a fragment of the Cytochrome c oxidase subunit I (COI) gene was carried out using the primers LepF1 (5′ATTCAACCAATCATAAAGATATTGG-3′), and LepR1 (5′TAAACTTCTGGATGTCCAAAAAATCA-3′) (Hebert et al. 2004). This COI barcode region was amplified via polymerase chain reaction (PCR) on a SENSQUEST Lab Cycler. Thermal PCR conditions were as follows: 95 °C for 5 min; 38 cycles of 95 °C for 30 s, 49 °C for 90 s, 72 °C for 60 s; 68 °C for 30 min. To confirm the correct amplification or detect undesired contaminations, PCR products were visualised using gel electrophoresis. Amplicons were purified with Thermo Scientific Exonuclease I, and FastAP Thermosensitive Alkaline Phosphatase Clean-up Kit. They were sequenced at Macrogen Europe with complements and sufficient overlap with adjacent regions to ensure the accuracy of the sequence data.

Sequence data were imported into Geneious R10 (https://www.geneious.com) for nucleotide editing and contig assembly. A multiple sequence alignment was performed using Bioedit 7.2.5 (Hall 1999) and then converted to Fasta and Nexus formatted files to use in different analysis programs. A 658 bp COI fragment was amplified for 23 individuals of *N.floweri* from three Iranian populations. All sequences are deposited in GenBank (https://www.ncbi.nlm.nih.gov/genbank/) with the following accession numbers: OQ223227–OQ223249. An additional 38 sequences of Oman specimens were obtained from GenBank under the accession numbers MK950279–MK950316. Information regarding the samples used in this study is provided in supporting information Suppl. material 1: table S2. In order to visualise the genetic relationships between different geographic populations, a haplotype network was constructed with the TCS network algorithm (Clement et al. 2002) implemented in the software PopART v. 1.7.2 (Leigh and Bryant 2015).

## ﻿Results

### ﻿Ecology

Individuals of *N.floweri* were observed resting on the leaves of the tree canopy as well as on grass in grasslands always at some distance from fresh water bodies. They have never been observed on the ground surface. According to our observations, it seems that this species is more active during night. In Iran, it prefers the hot and dry climate of the country’s southern and south-eastern parts. During our observations, two adults in their natural habitat, as well as three adults and two juveniles in captivity, preyed on small ants.

### ﻿Variation in male genitalia

**Figure 3.**
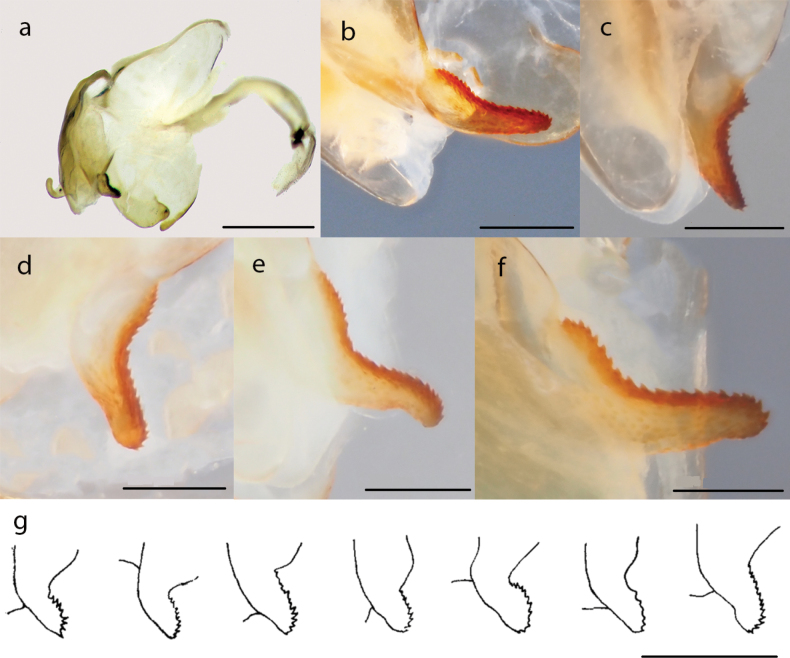
Male genitalia of *Nilomantisfloweri* and variability of the male apophysis phalloid: **a, b** Kangan (Iran) **c** Jam (Iran) **d** Bandar Lengeh (Iran) **e** Firooz Abad (Iran) **f** Chahbahar (Iran) **g** from right to left; specimens from Mauritania, Niger, Sennaar (Sudan) (type of *floweri*), Assab (Eritrea) (type of *tenella*), Eritrea, Jeddah and Mecca (Saudi Arabia). Scale bars: 1 mm (**a**), 300 µm (**b–g**). Fig. 2g is from Roy and Leston (1975) courtesy of Roger Roy (MHNN, Paris, France).

We examined the male genitalia of the specimens that were collected from various districts of Iran’s provinces (four males from Bandar-e Lengeh 27°13'26"N, 56°21'10"E, Hormozgan province; two males from Kangan 27°43'36"N, 52°12'34"E, two males from Jam 27°53'01"N, 52°21'16"E, Bushehr province; one male from Firuzabad 28°53'20"N, 52°33'07"E, Fars province; one male from Chabahar 25°17'40"N, 60°37'17"E, Sistan and Balochistan province). The phalloid apophysis in all specimens showed relatively high variability in shape and length within Iranian populations (Fig. 3).

### ﻿Ootheca morphology and life history

**Figure 4.**
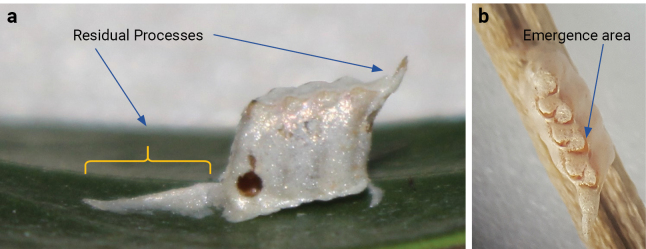
Ootheca of *Nilomantisfloweri*: **a** lateral view **b** dorsal view.

The ootheca of *N.floweri* is ovate (Fig. 4a, b), acuminate-like, and dorsally flattened. It has residual processes, on both ventral and dorsal sides (Fig. 4b). The colour of the ootheca is white or silvery. Four oothecae were obtained in this study. Two of them were collected from the natural habitat deposited on leaves of *Conocarpus* trees and the other two were laid by two of the females collected during field surveys. The largest ootheca contained eight eggs, the smallest three. The mean incubation duration for the oothecae was 43.5 days ± 1.7 under lab conditions (room temperature at 25 °C ± 2 with a 14 L: 10 D photoperiod cycle). More detailed and additional information (e.g., regarding size, and hatching numbers) is reported in Table 2. The first instar of *N.floweri* has a whitish colour with a delicate and slender body. After the first moult, the colour turns light green. After 68 days, only one out of the fourteen emerged nymphs reached adulthood. It moulted five times in order to reach maturity.

**Table 2.** Information regarding *Nilomantisfloweri* oothecae. Oothecae one and two were collected from their natural habitat, and three and four were laid in the lab, *n* = Number.

Ootheca	Width (mm)	Length (mm)	Incubation duration (days)	*n* eggs	*n* hatched nymphs
1	2.3	5.0	45	3	3
2	3.0	9.0	42	8	6
3	2.5	7.0	42	4	4
4	2.2	5.5	45	3	1
Mean	2.5	6.6	43.5	4.5	3.5
SD	0.4	1.8	1.7	2.4	2.1

### ﻿Distribution

**Figure 5.**
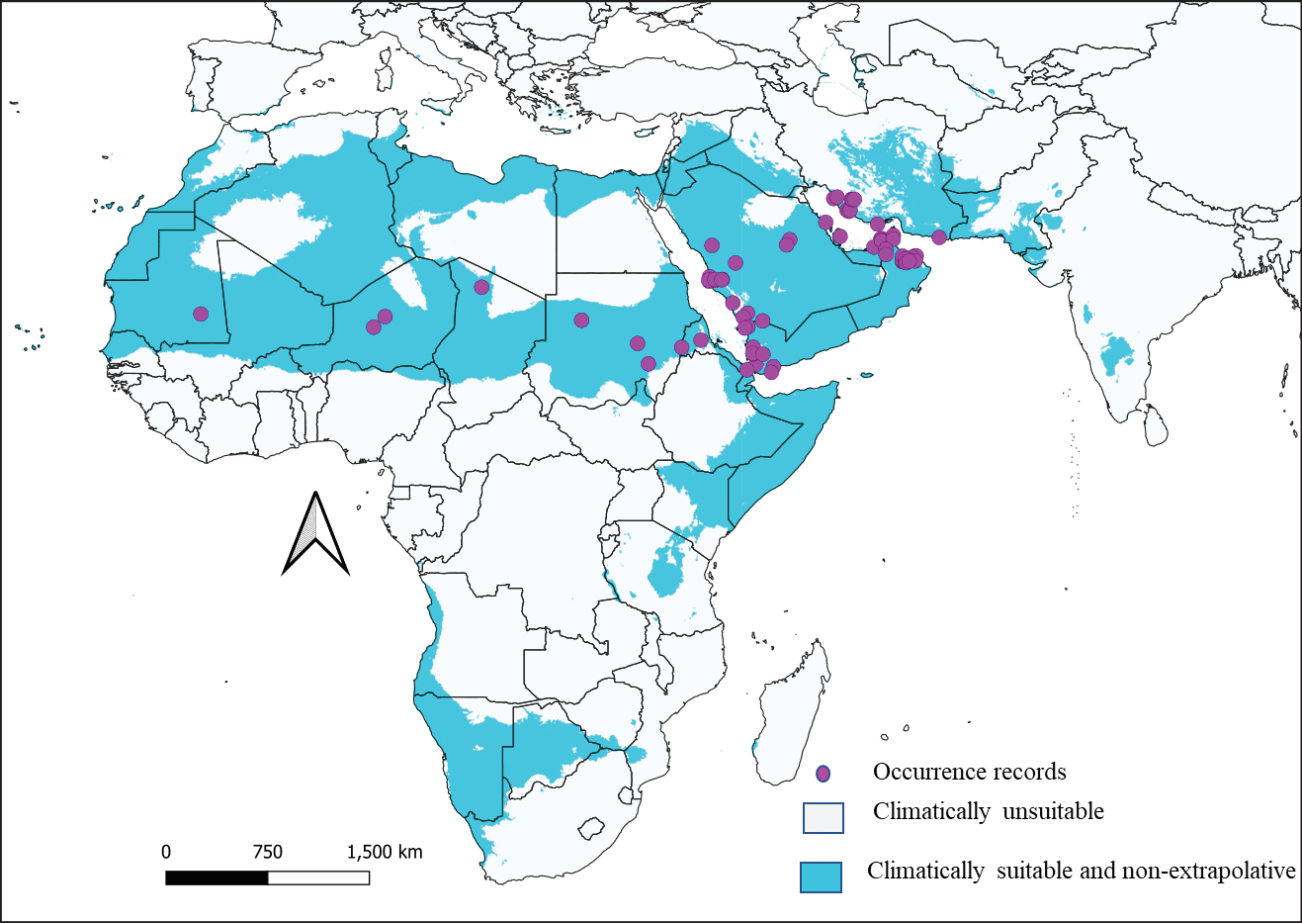
Final climate suitability map showing the current climatic suitability with a 10% threshold for *N.floweri* in its native range, visualisation with QGIS v. 3.22.

The known native distribution of this species includes tropical deserts, semi-deserts, and thorn savannahs of the Sahel, Arabia, and southern Iran. A total of 206 specimens from 164 locations with different coordinates of *N.floweri* was examined, with distribution records obtained from countries in North Africa (Mauritania, Niger, Chad, Sudan, and Eritrea), Saudi Arabia, Yemen, Oman, the United Arab Emirates, and Iran (Table 1). Specimens from North Africa were mostly recorded in areas far away from coastal regions, in arid thorn savannah of the Sahel. All these specimens from the Sahel were collected in 1897–1958. Other known records of *N.floweri* are mostly associated with coastal areas in south-western Asia, the large majority of which are distributed along the coasts of the Red Sea, but also the Persian and Oman Gulf. The oldest record from these areas belong to a male and a female documented in 1901 from Iran, while the most recent records belong to specimens from Dubai (United Arab Emirates) reported in 2022. All known records of *N.floweri*, based on both literature and new collection data published here, are illustrated in Fig. 5. According to the data herein examined, Yemen, Oman, Saudi Arabia, and Iran are the countries where this species has been recorded more frequently, whereas Sahel countries appear to have the lowest number of published records (Table 1).

### ﻿Ecological niche modelling

The configurations selected to produce the final model included environmental ‘set 3’, regularisation multiplier 0.5, and linear feature classes (Suppl. material 1: table S3). The spatial distribution model under the current climate condition showed discriminative performance in predicting the distribution of *N.floweri* (Fig. 5), while the model corresponded to the known distribution of this species from the Sahel zone in North Africa to the Arabian Peninsula and Iran. Partial ROC and area under the curve (AUC) values showed that the final model was significantly better than random expectations (*P* < 0.001; mean AUC = 1.079; omission rate at 5% = 0.05).

In addition to the areas where *N.floweri* has been recorded, our model expands the putatively climatically suitable areas for *N.floweri* beyond the regions where it has been recorded. This area includes dry and desert regions such as the Gobi Desert in China and Mongolia, the west and south parts of Pakistan, India, Iran’s eastern and central parts, and the northern and southern parts of Africa (Suppl. material 1: fig. S1). Areas of extrapolation of the current models were detected in the Gobi Desert in China and Mongolia (Suppl. material 1: fig. S2) and excluded from the final model (Fig. 5).

**Figure 6.**
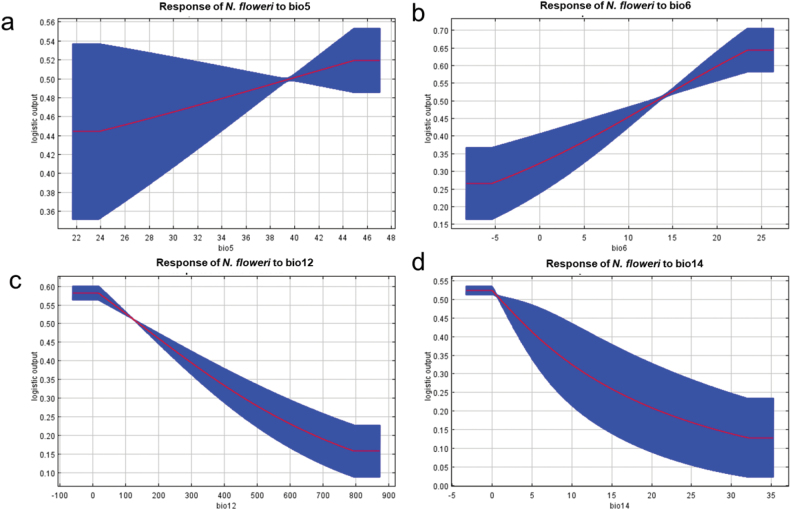
Response curves of the most relevant environmental factors affecting the distribution of *Nilomantisfloweri*; the shown values are an average of ten replicate runs **a** maximum temperature of the warmest month (Bio5) **b** minimum temperature of the coldest month (Bio6) **c** annual precipitation (Bio12) **d** precipitation of driest month (Bio14).

The annual precipitation (Bio12) and minimum temperature of the coldest month (Bio6) had the highest percentage of contribution in predicting the climate niche of *N.floweri* across its native range, followed by precipitation of the driest month (Bio14) and maximum temperature of the warmest month (Bio5) (Table 3). The response curves of the annual precipitation and precipitation of the driest month showed the climatic suitability of *N.floweri* when the annual precipitation was less than 100 mm and the precipitation of the driest month was less than 5 mm (Fig. 6). The response curves for the maximum temperature of the warmest month showed a linear increase with the increase in temperature, and the response curves for the minimum temperature of the coldest month showed a linear increase in the habitat suitability of *N.floweri* when the minimum temperature of the coldest month is between 15 and 24 °C (Fig. 6).

**Table 3.** Summary of bioclimatic predictors and their relative importance (in %) to model habitat suitability of *Nilomantisfloweri* across its native range. The pairwise correlation among these layers was less than 0.8.

Abbreviation	Variable	Units	Percent contribution
Bio 5	Max temperature of warmest month	°C	13.2
Bio 6	Minimum temperature of coldest month	°C	33.6
Bio 12	annual precipitation	mm/a	38.1
Bio 14	Precipitation of driest month	mm/month	15.1

### ﻿Barcodes and haplotype network

**Figure 7.**
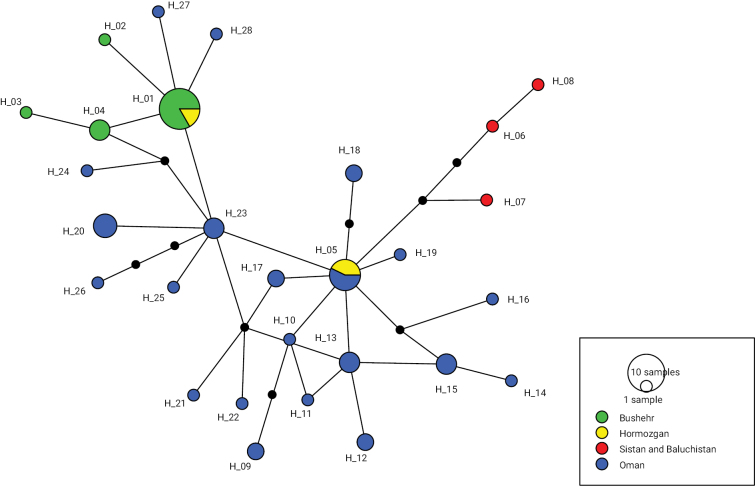
Haplotype network of *Nilomantisfloweri* based on the COI gene fragment. Circle sizes are proportional to haplotype frequency, black dots represent missing haplotypes. Colours refer to different localities. Largest circle indicates 10 samples and smallest 1 sample.

**Figure 8.**
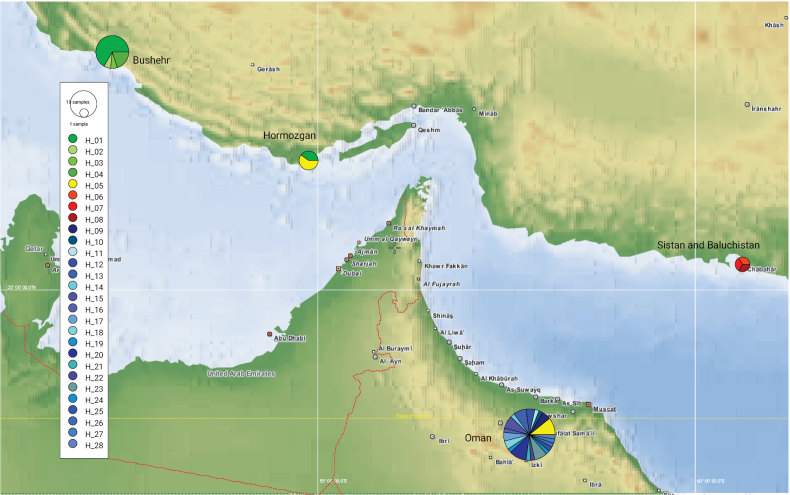
Geographic distribution of the 28 COI haplotypes recorded for *Nilomantisfloweri*.

Our haplotype analysis of the 60 sequences resulted in a single network consisting of 28 distinct haplotypes (Suppl. material 1: table S2). Apart from one haplotype (H_05) which was shared among specimens from Oman and Iran, the other were unique for either Oman (20) or Iran (7). The resulting network tree (Fig. 7) supports a close relationship between the Iranian and Oman haplotypes, being separated by only one to two mutational steps. Haplotype H_05, shared by both countries, included three sequences from Bandar-e Lengeh (Hormozgan Province, Iran), and five sequences from Oman. In contrast to the haplotypes found in Bushehr and Hormozgan which are part of the main network, the ones from Sistan and Baluchistan Province in south-eastern Iran (Fig. 8) form a slightly distinct clade.

## ﻿Discussion

### ﻿Taxonomic remarks

The genus *Nilomantis* and its type species *Nilomantisfloweri* were described by Werner (1907), based on a single male specimen collected in Sennaar, Sudan. The rounded shape of the compound eyes, the keeled pronotum, and the “flattened” head capsule were emphasised as important diagnostic characteristics for this species. Giglio-Tos (1915) described a new genus and species of praying mantis, *Cryptomantistenella* Giglio-Tos, 1915, from Katona (East Africa), which externally resembled *Nilomantis* but was distinct from the latter genus by having conical compound eyes. Beier (1930) described another *Nilomantis* species, *N.arabica* Beier, 1930, from Muscat (Oman). He stated that *N.arabica* was distinguished from the previously described *N.floweri* and *C.tenella* by its larger body size and the presence of two reddish brown spots between the ocelli and the compound eyes. Beier (1930) also examined the type of *N.floweri* claiming that the rounded compound eyes reported by Werner (1907) for this species were actually conical and thus not corresponding to the author’s original description. Since the only generic difference between *Nilomantis* and *Cryptomantis* was the shape of the eyes, Beier (1930) transferred *Cryptomantistenella* to the genus *Nilomantis*.

In his publication on the Arabian Mantodea, Uvarov (1939) stated that *N.floweri* was a common species in Saudi Arabia, as evidenced by the specimens collected from various localities in this country (i.e., Jidda, Mecca, Taif, Midakhil, Riyadh, Khamis Mushait). According to his examination, all the specimens collected were uniform in size and coloration and showed no differences in comparison with specimens from Sudan. He also mentioned that *N.arabica* described by Beier (1930) was noticeably larger than *N.floweri*, but the two red spots on the vertex that Beier (1930) considered as a distinguishing feature between the two species were sometimes present in *N.floweri* as well. Due to the discovery of *Nilomantis* specimens in Ghana, Roy and Leston (1975) described another *Nilomantis* species, *N.edmundsi* Roy & Leston, 1975. They examined all type specimens of this genus and, based on the comparison of male genitalia and geographic distribution of the species, they synonymised *C.tenella* and *N.arabica* with *N.floweri*. They also provided a list of known specimens from various countries.

Kaltenbach (1982) also recorded *N.floweri* from different localities in Saudi Arabia. In addition, he mentioned the presence of this species in south-eastern Iran (Baluchistan), without providing any proof for Iranian records (i.e., deposition of Iranian specimens). In his book on the Mantodea of the world, Ehrmann (2002) further confirmed the presence of *N.floweri* in Iran, but again without reporting any detail or information regarding the deposition of the specimens. Thus, prior to this publication, the occurrence of this species in Iran had to be considered uncertain.

### ﻿Variation in male genitalia

Despite the high morphological variability that we observed in the shape of the phalloid apophysis (Fig. 3), the genetic distance among populations is low, and thus we confirm that this is just intraspecific variability. A correlation between male genitalia morphology and successful fertilisation has been shown in some studies to illustrate the impact of sexual selection on genital morphology (Otronen 1998; Arnqvist and Danielsson 1999; House and Simmons 2003; Rodriguez et al. 2004; Wenninger and Averill 2006). Holwell et al. (2010) studied the male genitalia morphology and its correlation with sperm transfer success in the praying mantis *Ciulfinaklassi* Holwell et al. 2007. They reported that the variation in male genitalia in this mantid species may be due to the different sperm diffusing ways to the female spermatheca. Their research strongly implies that complex male genital structures of *C.klassi* have in fact been shaped for effective sperm transfer, possibly as a result of sperm competition. This is also the most parsimonious explanation for the patterns observed in our study. Therefore, postcopulatory female choice might have resulted in the observed pattern of high morphological variability in *N.floweri* as a consequence of female responses to variations in the shape of male genitalia.

### ﻿Ecology and life history

*Nilomantisfloweri* represents a unique ecological exception among the Nanomantidae family by preferentially inhabiting arid Sahelian areas. The members of this family are predominantly associated with moist subtropical and tropical regions (Brannoch and Svenson 2019). However, most of the species belonging to the Nilomantini tribe (sensu Schwarz and Roy 2019) primarily occupy savanna environments with some presence in rainforest regions across tropical Africa. Even *N.edmundsi*, the sister species of *N.floweri*, is predominantly found in Guinea savanna.

Our knowledge on the ecology, biology, and habitat preferences of *N.floweri*, and in general of the entire Nanomantidae family, is very limited. Such information is crucial to identify their conservation status and the response to climate change and should be addressed. According to studies on life cycles of some mantid families, the size, quantity, and quality of food consumed by the female can impact the size of the oothecae and the number of eggs per ootheca in mantids (Birkhead et al. 1988; Mirzaee et al. 2022). This also seems to be true for *N.floweri*, which has a small size (15–21 mm) resulting in small oothecae with few eggs. In contrast, large-sized mantids such as *Hierodulatenuidentata* Saussure, 1869 and *Mantisreligiosa* (Linne, 1758) have much larger ootheca, with 100 to 200 eggs per ootheca (Fagan and Hurd 1994; Mirzaee et al. 2022). As *N.floweri* is well capable of colonising extremely anthropogenic habitats, its habitat in Iran is very diverse, ranging from completely man-made places to natural environments. However, all of these habitats share the presence of water (i.e., rivers, or natural spring, places close to the sea shore), vegetation (i.e., *Rhus*, *Acacia*) and are all located in Iran’s semi-desertic zone in the southern and south-eastern parts.

#### ﻿Biogeography of *N.floweri* and origin of Iranian populations

Our research confirms the presence of *N.floweri* in Iran and shows that the native range of this species covers all major parts of the Saharo-Arabian realm sensu Holt et al. (2013). Due to the small number of faunal elements restricted to this area, the Saharo-Arabian realm was previously seen mainly as a transitional area between the Palearctic and the Afrotropical realm (e.g., Müller 1981). The distribution of *N.floweri* reported in this study is in contrast with this view and instead supports the one of Holt et al. (2013). Based on the known records, *N.floweri* is apparently mostly associated with coastal areas in south-western Asia, with the large majority of records being distributed along the coasts of the Red Sea and the Persian and Oman Gulf. The commonness in its Arabian distribution is in strong contrast with the records of *N.floweri* in North Africa where most of the collections are reported in the transitional zone between the southern Sahara and the arid thorn savannah, far off the coast (Fig. 5). Perhaps this very limited distribution in the transitional zone between the southern Sahara and the arid thorn savannah might be due to the intraguild predation, particularly resource competition. This competition serves as a major factor influencing population sizes in such regions due to a lower level of competition, since only a small number of animals and insects can survive in the harsh environmental conditions. As a result, *N.floweri* may have occupied a specific ecological niche that is less competitive, leading to its restricted distribution in this particular region. In North Africa *N.floweri* seems to live in mountainous areas, near water sources, and even anthropogenic places with a minimum of vegetation, which are providing insect prey as a food resource.

Regarding the distribution pattern of *N.floweri* in Iran and their genetic composition, a natural spreading from the Arabian Peninsula to Iran is plausible. Today, geographic barriers (i.e., the Persian Gulf and the Gulf of Oman) are separating populations of these areas, limiting gene flow between them. On the other hand, these barriers are not constant, allowing exchange among populations, as suggested by their close relationships and the limited number of mutations between their haplotypes. Thus, the Gulf of Oman does not appear as a strong biogeographic barrier for this species since populations living on different sides of the gulf appear directly interconnected by even owing one identical haplotype. Such a close relationship suggests that their separation may have occurred relatively recently or that the current distribution is even the result of human transport. Additionally, the presence of two distinct and genetically distant groups of populations within Iran suggests multiple colonisation events. To further investigate this hypothesis, it is necessary to conduct future studies utilising more comprehensive data, such as increasing the number of samples from populations across its range or employing additional genetic markers. It is highly probable that these events occurred during glacial periods when lower sea levels caused significant desiccation of the Persian Gulf and substantial reduction in the size of the Gulf of Oman (Uchupi et al. 1999).

### ﻿Ecological niche modelling

The climatically suitable areas recovered in our distributional models closely correspond to the known distribution of *N.floweri* (Fig. 5, Table 1). It seems that the areas recovered as suitable for *N.floweri* are mostly arid regions with some access to water sources, which is important for the moulting process of the species (Yager 1999). However, the presence of at least some vegetation is also crucial for the survival of the species, as it provides camouflage and protection from predators. It is common for many insect species to have specific habitat requirements, and *N.floweri* is no exception. By tracing the species’ ecological needs, we can better understand where it is likely to be found and what measures can be taken to protect it.

Our final climate niche model (Fig. 5) also recovered climatically suitable areas within countries with no previous record of the species (i.e., Pakistan). This could represent sampling gaps, as these regions share the climatic profile and landscape features known in *N.floweri*’s distribution. In southern Pakistan, for instance, the climate is generally hot and dry, and shows similar vegetation features as the one found within its native range such as *Acacia* trees and other desert shrubland species. This suggests that the species could potentially survive and even thrive in these areas, although further research is needed to confirm the presence of the species and the extent of their potential habitat.

MOP analysis (Suppl. material 1: fig. S2) indicate areas of extrapolation such as the Gobi Desert in China and Mongolia, with high temperature fluctuations, both daily and annually (Pfeiffer et al. 2003). While it is possible that some species may be able to adapt to such extreme conditions over time, it is unlikely that *N.floweri* would be able to survive in such an environment, given its specific temperature requirements. *Nilomantisfloweri* can only inhabit places with no real winter with minimum temperatures not below 5 to 8 °C. Therefore, it cannot survive in these areas due to the extremely cold temperatures (a mean of often less than -15 °C). However, indication of climatic suitability of these winter-cold desert areas seems to be due to the inadequate model for the coldest month (Fig. 6b), assuming a logistic output of > 0.25 even for temperatures below zero.

It is likely that the climatic conditions in southern Africa are suitable for *N.floweri*, as temperature and precipitation well fall within its required range. However, this area is geographically far away from the present distribution of the species so that absence just might be explained by physical distance. Furthermore, the forests of Central and Eastern Africa, separating Africa’s northern and southern regions (Adams 1998; Congo Basin Forest Partnership 2005), may additionally have served as a geographical barrier. They have limited the ability of species to move between these regions and adapt to different environmental conditions. Thus, large herbivores of the savannah biome are often split into northern and southern phylogroups (Lorenzen et al. 2012). However, biotic conditions in this region may also be unsuitable for this species to survive. This could include factors such as competition with other species, lack of suitable food sources, or predation by other animals.

In addition, species can adapt and evolve over time to overcome barriers and expand their range. Understanding the potential for such adaptations and the mechanisms by which it occurs is an important area of research for predicting and managing species distributions in a changing climate (Nadeau and Urban 2019). In our study, we only considered climatic factors and the distribution records in order to have a better understanding of the pattern of the distribution of *N.floweri*; however, future studies should include landscape features, which could potentially shed light on distributional barriers hindering species expansion into areas recovered as climatically suitable in our study.

## ﻿Conclusions

Our study sheds new light on the knowledge of this small mantid species, summarising all the published records and reporting new geographic data and information on its ecology based on rearing and newly collected specimens from Iran. Nevertheless, our knowledge of *N.floweri* is still far from complete. In this regard, the lack of recent records and genetic data for several countries within the supposed range of the species does not allow us to fully understand the distribution and ecology of *N.floweri* nor its historical biogeography. In particular, the contrasting patterns of distribution and habitat preference in populations from south-western Asia and North Africa remain unresolved. Additional collections in poorly surveyed areas would help to fill these gaps in the future and would also help to determine the conservation status of this Sahelian mantid species.

## ﻿Additional information

### Author ORCIDs

Zohreh Mirzaee https://orcid.org/0000-0002-1496-8253

Saber Sadeghi https://orcid.org/0000-0002-3183-8979

Francesco Ballarin https://orcid.org/0000-0003-1417-2519

Thomas Schmitt https://orcid.org/0000-0002-1389-8396

Marianna Simões https://orcid.org/0000-0003-4401-5530

Martin Wiemers https://orcid.org/0000-0001-5272-3903

### Data availability

All of the data that support the findings of this study are available in the main text or Supplementary Information.

## Supplementary materials

Supplementary material 1 New insights into the life history, ecological niche modeling, and biogeography of the enigmatic mantid *Nilomantisfloweri* (Mantodea, Nanomantidae) Data type occurences, Modelling data, maps Explanation note This document contains maps that display the present climate suitability, accession numbers of samples, information on sample occurrences, and other relevant data utilized in this research. Additionally, it includes data on the performance of 84 different models. https://binary.pensoft.net/file/888359This dataset is made available under the Open Database License (http://opendatacommons.org/licenses/odbl/1.0/). The Open Database License (ODbL) is a license agreement intended to allow users to freely share, modify, and use this Dataset while maintaining this same freedom for others, provided that the original source and author(s) are credited.Zohreh Mirzaee, Saber Sadeghi, Francesco Ballarin, Thomas Schmitt, Marianna Simões, Martin Wiemers
