# Supplementary material for: ﻿Life history and biogeography of the enigmatic mantid Nilomantisfloweri (Mantodea, Nanomantidae)
Source: Zookeys. 2023 Aug 4;1173:275–95. doi: 10.3897/zookeys.1173.107204 (PMC10422127; doi:10.3897/zookeys.1173.107204)
Supplement: Supplementary material 1 — New insights into the life history, ecological niche modeling, and biogeography of the enigmatic mantid Nilomantisfloweri (Mantodea, Nanomantidae) [file zookeys-1173-275_article-107204__-s001.docx]

Supporting Information

**New insights into the life history, ecological niche modeling, and biogeography of the enigmatic mantid *Nilomantis floweri* (Mantodea, Nanomantidae)**

Zohreh Mirzaee, Saber Sadeghi, Francesco Ballarin, Thomas Schmitt, Marianna Simões, Martin Wiemers

**Supplementary Figure S1:** Map showing the current climatic suitability before MOP analysis, with a 10 % threshold for *N. floweri*, visualization with QGIS v. 3.22. Blue indicates areas of high climatic suitability, whereas white represents areas with less favourable climatic conditions.

**
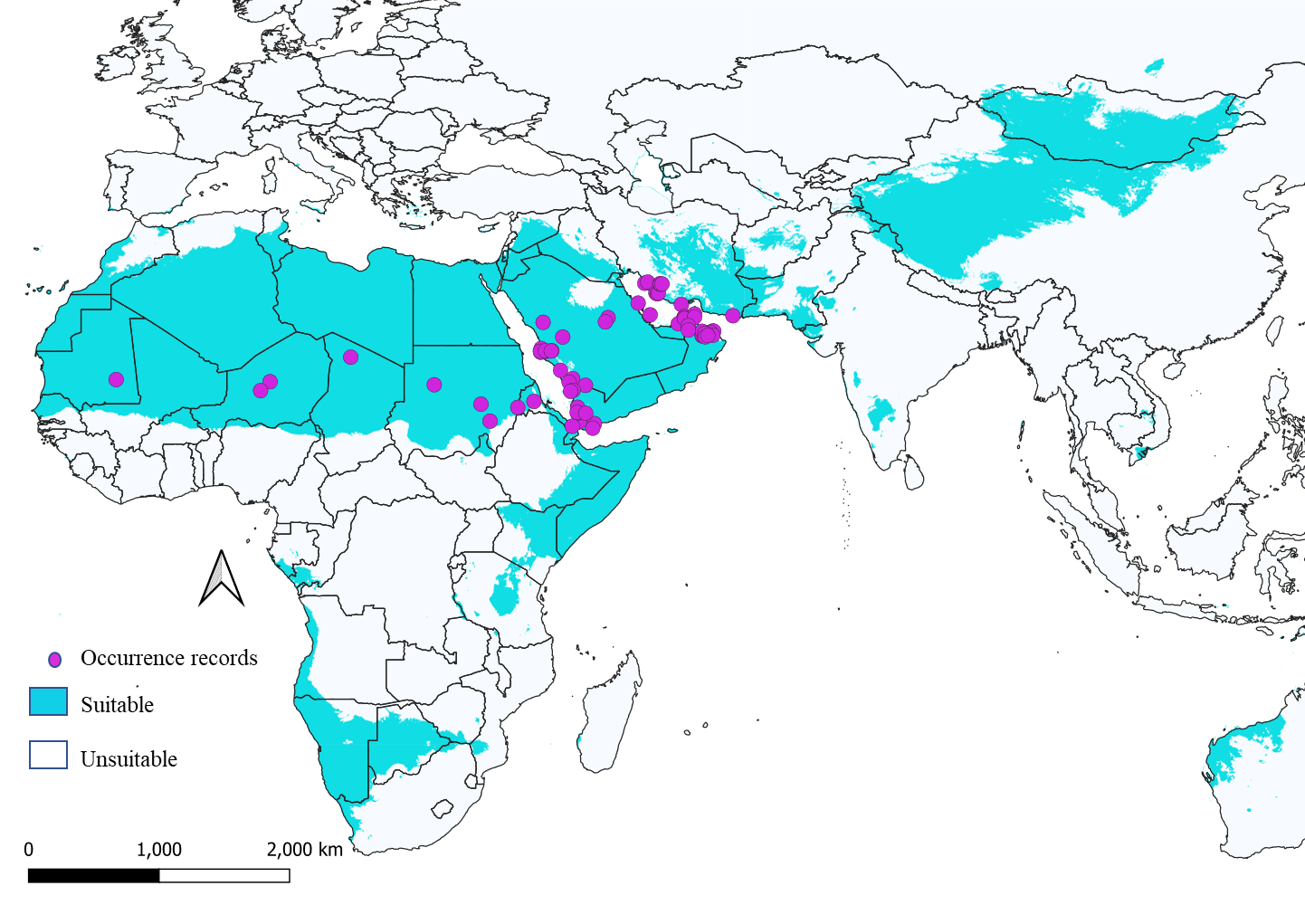
**

**Supplementary Figure S2:** Map showing the model extrapolation area obtained from MOP analysis. Blue areas show extrapolative regions. Non-extrapolative areas are shown in white.

**
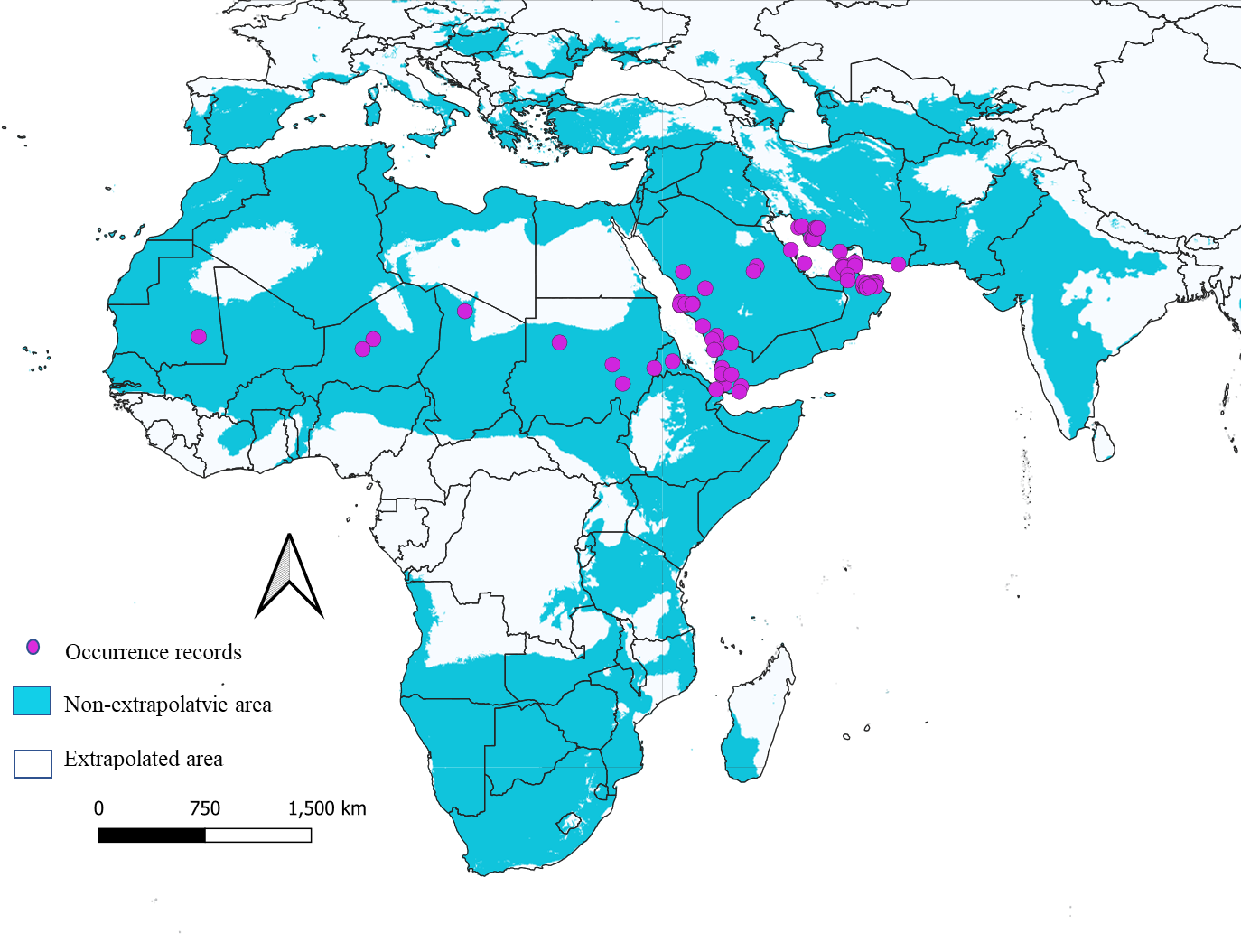
**

**Supplementary Table S1:** Sample localities of *N. floweri*.

| **Species** | **Latitude** | **Longitude** | **Source** | **Year** | **City, Province, Country** | **NOF** | **Sex** |
| --- | --- | --- | --- | --- | --- | --- | --- |
| *N. floweri* | 28.914888 | 50.815553 | This study | 18.3.2021 | Bushehr, Bushehr, Iran | 1 | 1 male |
| *N. floweri* | 28.918857 | 50.810896 | This study | 18.3.2021 | Bushehr, Bushehr, Iran | 1 | 1 female |
| *N. floweri* | 28.925615 | 50.808286 | This study | 18.3.2021 | Bushehr, Bushehr, Iran | 1 | 1 female |
| *N. floweri* | 29.064738 | 51.127071 | This study | 17.4.2020 | Khormuj, Bushehr, Iran | 1 | 1 female |
| *N. floweri* | 29.061112 | 51.131028 | This study | 17.4.2020 | Khormuj, Bushehr, Iran | 1 | 1 female |
| *N. floweri* | 27.837874 | 52.048782 | This study | 3.4.2018 | Kangan, Bushher, Iran | 2 | 1 male  1 nymph |
| *N. floweri* | 27.835588 | 52.052483 | This study | 3.4.2018 | Kangan, Bushher, Iran | 1 | 1 female |
| *N. floweri* | 27.845478 | 52.084887 | This study | 3.4.2018 | Kangan, Bushher, Iran | 1 | 1 female |
| *N. floweri* | 27.735541 | 52.202416 | This study | 15.4.2019 | Tombak, Kangan, Bushher, Iran | 1 | 1 male |
| *N. floweri* | 27.735636 | 52.200603 | This study | 15.4.2019 | Tombak, Kangan, Bushher, Iran | 1 | 1 male |
| *N. floweri* | 27.735826 | 52.198468 | This study | 15.4.2019 | Tombak, Kangan, Bushher, Iran | 1 | 1 male |
| *N. floweri* | 27.735232 | 52.198509 | This study | 15.4.2019 | Tombak, Kangan, Bushher, Iran | 1 | 1 female |
| *N. floweri* | 27.815288 | 52.330418 | This study | 20.3.2017 | Jam, Bushher, Iran | 1 | 1 female |
| *N. floweri* | 27.820616 | 52.333573 | This study | 5.4.2019 | Jam, Bushher, Iran | 2 | 2 females |
| *N. floweri* | 27.82073 | 52.331942 | This study | 5.4.2019 | Jam, Bushher, Iran | 2 | 2 nymphs |
| *N. floweri* | 27.818744 | 52.332991 | This study | 17.3.2020 | Jam, Bushher, Iran | 1 | 1 male |
| *N. floweri* | 27.829634 | 52.331003 | This study | 14.42021 | Jam, Bushher, Iran | 1 | 1 female |
| *N. floweri* | 28.864739 | 52.474005 | This study | 3.5.2020 | Firoz Abad, Fars Iran | 1 | 1 male |
| *N. floweri* | 28.818015 | 52.566939 | This study | 3.5.2020 | Firoz Abad, Fars Iran | 1 | 1 female |
| *N. floweri* | 28.854614 | 52.737744 | This study | 8.5.2021 | Meymand, Fars Iran | 1 | 1 female |
| *N. floweri* | 28.847916 | 52.73423 | This study | 8.5.2021 | Meymand, Fars Iran |  | 1 female |
| *N. floweri* | 26.544378 | 54.877843 | This study | 12.4.2021 | Bandar Lengeh, Bandar Abbas, Iran | 1 | 1 female |
| *N. floweri* | 26.545827 | 54.879678 | This study | 12.4.2021 | Bandar Lengeh, Bandar Abbas, Iran | 1 | 1 male |
| *N. floweri* | 26.544272 | 54.877011 | This study | 12.4.2021 | Bandar Lengeh, Bandar Abbas, Iran | 1 | 1 male |
| *N. floweri* | 26.554115 | 54.889141 | This study | 12.4.2021 | Bandar Lengeh, Bandar Abbas, Iran | 1 | 1 male |
| *N. floweri* | 26.555113 | 54.889592 | This study | 12.4.2021 | Bandar Lengeh, Bandar Abbas, Iran | 1 | 1 male |
| *N. floweri* | 25.301038 | 60.608279 | This study | 2.5.2020 | Chahbahar, Sistan va Baluchistan,  Iran | 1 | 1 male |
| *N. floweri* | 25.307856 | 60.62577 | This study | 2.5.2020 | Chahbahar, Sistan va Baluchistan,  Iran | 1 | 1 female |
| *N. floweri* | 25.306202 | 60.627439 | This study | 2.5.2020 | Chahbahar, Sistan va Baluchistan,  Iran | 1 | 1 female |
| *N. floweri* | 26.45 | 57.63333 | This study | 6-11.4.2000 | Dehan, Hormozgan, Iran | 1 | male |
| *N. floweri* | 60.627439 | 60.17222 | This study | 9.4.2000 | Nikshahr, Sistan va Baluchestan, Iran | 2 | males |
| *N. floweri* | 25.307717 | 60.633744 | This study | 28.3.1901 | Makran, Sistan va Baluchistan,  Iran | 2 | 1 male 1 female |
| *N. floweri* | 27.23444 | 57.0225 | This study | 6-17.4.2016 | Hormozgan, Minab Road, Iran | 1 | male |
| *N. floweri* | 23.06 | 57.21 | SMNK | 9. 2000 | Nizwa, Oman | 1 | 1 male |
| *N. floweri* | 25.10 | 46.71502 | SMNK | 28.8.1959 | Riyadh, Saudi Arabia | 2 | NA |
| *N. floweri* | 25.10 | 46.71502 | SMNK | 28.8.1959 | Riyadh, Saudi Arabia |  | NA |
| *N. floweri* | 14.53 | 43.26 | SMNK | 29.3.2000 | Al Hudaydah, Yemen | 1 | 1 male |
| *N. floweri* | 15.11 | 43.32 | SMNK | 3.11.1996 | Al-Mahwit, Yemen | 1 | NA |
| *N. floweri* | 15.11 | 43.32 | SMNK | 3.11.1996 | Al-Mahwit, Yemen | 1 | NA |
| *N. floweri* | 15.11 | 43.32 | SMNK | 3.11.1996 | Al-Mahwit, Yemen | 1 | NA |
| *N. floweri* | 15,12,98 | 43.3158 | SMNK | 22.4.1998 | Al-Mahwit, Yemen | 1 | 1 female |
| *N. floweri* | 15,12,98 | 43.3158 | SMNK | 22.4.1998 | Al-Mahwit, Yemen | 1 | 1 female |
| *N. floweri* | 13.53 | 43.58 | SMNK | 12.3.2000 | Ibb, Yemen | 1 | 1 male |
| *N. floweri* | 13,24,88 | 44.1485 | SMNK | 26.4.1998 | Ta'izz, Yemen | 1 | 1 male |
| *N. floweri* | 14.48 | 43.32 | SMNK | 9.1988 | Jebel Bura, Yemen | 1 | 1 male |
| *N. floweri* | 14.48 | 43.32 | SMNK | 9.1988 | Jebel Bura, Yemen | 1 | 1 female |
| *N. floweri* | 14.48 | 43.32 | SMNK | 10.5.1998 | Jebel Bura, Yemen | 1 | NA |
| *N. floweri* | 14.48 | 43.32 | SMNK | 10.5.1998 | Jebel Bura, Yemen | 1 | NA |
| *N. floweri* | 14.48 | 43.32 | SMNK | 10.5.1998 | Jebel Bura, Yemen | 1 | NA |
| *N. floweri* | 14.48 | 43.32 | SMNK | 10.5.1998 | Jebel Bura, Yemen | 1 | NA |
| *N. floweri* | 14.48 | 43.32 | SMNK | 10.5.1998 | Jebel Bura, Yemen | 1 | NA |
| *N. floweri* | 14.48 | 43.32 | SMNK | 10.5.1998 | Jebel Bura, Yemen | 1 | NA |
| *N. floweri* | 14.48 | 43.32 | SMNK | 10.5.1998 | Jebel Bura, Yemen | 1 | NA |
| *N. floweri* | 14.48 | 43.32 | SMNK | 10.5.1998 | Jebel Bura, Yemen | 1 | NA |
| *N. floweri* | 14.48 | 43.32 | SMNK | 10.5.1998 | Jebel Bura, Yemen | 1 | NA |
| *N. floweri* | 14.48 | 43.32 | SMNK | 10.5.1998 | Jebel Bura, Yemen | 1 | NA |
| *N. floweri* | 14.48 | 43.32 | SMNK | 10.5.1998 | Jebel Bura, Yemen | 1 | NA |
| *N. floweri* | 14.48 | 43.32 | SMNK | 10.5.1998 | Jebel Bura, Yemen | 1 | NA |
| *N. floweri* | 14.48 | 43.32 | SMNK | 10.5.1998 | Jebel Bura, Yemen | 1 | NA |
| *N. floweri* | 14.48 | 43.32 | SMNK | 10.5.1998 | Jebel Bura, Yemen | 1 | NA |
| *N. floweri* | 14.54 | 43.27 | SMNK | 11.5.1998 | Jebel Bura, Yemen | 1 | NA |
| *N. floweri* | 14.54 | 43.27 | SMNK | 11.5.1998 | Jebel Bura, Yemen | 1 | NA |
| *N. floweri* | 14.54 | 43.27 | SMNK | 11.5.1998 | Jebel Bura, Yemen | 1 | NA |
| *N. floweri* | 14.54 | 43.27 | SMNK | 11.5.1998 | Jebel Bura, Yemen | 1 | NA |
| *N. floweri* | 14.54 | 43.27 | SMNK | 11.5.1998 | Jebel Bura, Yemen | 1 | NA |
| *N. floweri* | 14.54 | 43.27 | SMNK | 11.5.1998 | Jebel Bura, Yemen | 1 | NA |
| *N. floweri* | 14.54 | 43.27 | SMNK | 11.5.1998 | Jebel Bura, Yemen | 1 | NA |
| *N. floweri* | 14.54 | 43.27 | SMNK | 11.5.1998 | Jebel Bura, Yemen | 1 | NA |
| *N. floweri* | 14.54 | 43.27 | SMNK | 11.5.1998 | Jebel Bura, Yemen | 1 | NA |
| *N. floweri* | 14.54 | 43.27 | SMNK | 11.5.1998 | Jebel Bura, Yemen | 1 | NA |
| *N. floweri* | 14.54 | 43.27 | SMNK | 11.5.1998 | Jebel Bura, Yemen | 1 | NA |
| *N. floweri* | 14.53 | 43.27 | SMNK | 11.5.1998 | Jebel Bura, Yemen | 1 | NA |
| *N. floweri* | 14.53 | 43.27 | SMNK | 11.5.1998 | Jebel Bura, Yemen | 1 | NA |
| *N. floweri* | 14.53 | 43.27 | SMNK | 14.4.1997 | Jebel Bura, Yemen | 1 | 1 male |
| *N. floweri* | 14.53 | 43.27 | SMNK | 16.4.1997 | Jebel Bura, Yemen | 1 | 1 female |
| *N. floweri* | 13.30 | 45.2 | SMNK | 22.5.1998 | Zinjibar/Abyan, Yemen | 1 | 1 male |
| *N. floweri* | 13.30 | 45.2 | SMNK | 22.5.1998 | Zinjibar/Abyan, Yemen | 1 | 1 female |
| *N. floweri* | 14.46 | 44.23 | SMNK | 9.1988 | Dhamar, Yemen | 1 | 1 male |
| *N. floweri* | 14.46 | 44.23 | SMNK | 9.1988 | Dhamar, Yemen | 1 | 1 female |
| *N. floweri* | 25.126133 | 55.183115 | [iNaturalist](https://www.inaturalist.org/observations/127641921) | 20.7.2022 | Al Sufouh, Dubai, United Arab Emirates | 1 | NA |
| *N. floweri* | 25.12233 | 55.181833 | [iNaturalist](https://inaturalist.nz/observations/126844058) | 18.7.2022 | Al Sufouh, Dubai, United Arab Emirates | 2 | NA |
| *N. floweri* | 25.222751 | 55.260973 | [iNaturalist](https://www.inaturalist.org/observations/124549770) | 3.7.2022 | Bur Dubai, Dubai,  United Arab Emirates | 3 | NA |
| *N. floweri* | 23.597817 | 58.384098 | [iNaturalist](https://inaturalist.nz/observations/104279922) | 3.1.2022 | Muscat, Oman | 4 | NA |
| *N. floweri* | 26.724492 | 50.069031 | [iNaturalist](https://inaturalist.nz/observations/84485366) | 25.6.2021 | Qurtubah, Ras Tanura,  Saudi Arabia | 2 | NA |
| *N. floweri* | 25.029195 | 55.280249 | [iNaturalist](https://inaturalist.nz/observations/67608528) | 5.1.2021 | Wadi Al Safa 7, Dubai,  United Arab Emirates | 1 | 1 female |
| *N. floweri* | 24.904198 | 55.203097 | [iNaturalist](https://inaturalist.nz/observations/66088837) | 27.12.2020 | Dubai, United Arab Emirates | 2 | NA |
| *N. floweri* | 24.413867 | 54.535767 | [iNaturalist](https://www.inaturalist.org/observations/66768255) | 19.12.2020 | Khalifa City - Abu Dhabi,   United Arab Emirates | 2 | NA |
| *N. floweri* | 25.039727 | 55.270164 | [iNaturalist](https://inaturalist.nz/observations/66088837) | 4.12.2020 | Dubai, United Arab Emirates | 3 | NA |
| *N. floweri* | 25.408262 | 51.409725 | [iNaturalist](https://inaturalist.nz/observations/64653754) | 11.11.2020 | Umm Salal Municipality, Qatar | 1 | NA |
| *N. floweri* | 25.500332 | 56.361239 | [iNaturalist](https://www.inaturalist.org/observations/63751937) | 28.5.2019 | Fujairah, Saudi Arabia | 3 | 1 male 1 female 1 nymph |
| *N. floweri* | 25.501144 | 56.362544 | [iNaturalist](https://www.inaturalist.org/observations/63751825) | 26.5.2019 | Fujairah, Saudi Arabia | 2 | 1 male 1 female |
| *N. floweri* | 25.052989 | 55.261683 | [iNaturalist](https://www.inaturalist.org/observations/54491546) | 27.7.2020 | Wadi Al Safa 6, Dubai,  United Arab Emirates | 1 | NA |
| *N. floweri* | 25.002536 | 55.254375 | [iNaturalist](https://inaturalist.nz/observations/47709386) | 28.5.2020 | Al Hebiah Fifth, Dubai,  United Arab Emirates | 1 | NA |
| *N. floweri* | 23.602606 | 58.492058 | [iNaturalist](https://www.inaturalist.org/observations/43005128) | 5.4.2020 | Al Wutayyah, Muscat, Oman | 1 | NA |
| *N. floweri* | 25.145198 | 56.352322 | [iNaturalist](https://www.inaturalist.org/observations/34023478) | 3.1.2017 | Fujairah, Saudi Arabia | 1 | NA |
| *N. floweri* | 26.720151 | 50.075966 | [iNaturalist](https://www.inaturalist.org/observations/25375058) | 19.5.2019 | Ras Tanura, Saudi Arabia | 1 | NA |
| *N. floweri* | 24.213967 | 55.655357 | [iNaturalist](https://inaturalist.nz/observations/1467522) | 9.5.2015 | Abu Dhabi, United Arab Emirates | 1 | 1 male |
| *N. floweri* | 25.12952 | 55.18429 | [iNaturalist](https://inaturalist.nz/observations/1346159) | 14.11.2014 | Madinat al-Jumeirah, Dubai,  United Arab Emirates | 1 | NA |
| *N. floweri* | 23.0500 | 57.4600 | [GBIF](https://www.gbif.org/occurrence/3351624907) | 3/6/2017 | Oman | 1 | NA |
| *N. floweri* | 23.1500 | 57.2100 | [GBIF](https://www.gbif.org/occurrence/3351535921) | 3/6/2017 | Oman | 1 | NA |
| *N. floweri* | 23.0500 | 57.4600 | [GBIF](https://www.gbif.org/occurrence/3351533886) | 3/6/2017 | Oman | 1 | NA |
| *N. floweri* | 23.0500 | 57.4600 | [GBIF](https://www.gbif.org/occurrence/3349354831) | 3/6/2017 | Oman | 1 | NA |
| *N. floweri* | 23.1500 | 57.2100 | [GBIF](https://www.gbif.org/occurrence/3349354830) | 3/6/2017 | Oman | 1 | NA |
| *N. floweri* | 23.1500 | 57.2100 | [GBIF](https://www.gbif.org/occurrence/3349275518) | 3/6/2017 | Oman | 1 | NA |
| *N. floweri* | 22.9500 | 57.5400 | [GBIF](https://www.gbif.org/occurrence/3349253931) | 3/6/2017 | Oman | 1 | NA |
| *N. floweri* | 23.1500 | 57.2100 | [GBIF](https://www.gbif.org/occurrence/3349235626) | 3/6/2017 | Oman | 1 | NA |
| *N. floweri* | 23.1500 | 57.2100 | [GBIF](https://www.gbif.org/occurrence/3349210504) | 3/6/2017 | Oman | 1 | NA |
| *N. floweri* | 23.1500 | 57.2100 | [GBIF](https://www.gbif.org/occurrence/3349175707) | 3/6/2017 | Oman | 1 | NA |
| *N. floweri* | 22.9500 | 57.5400 | [GBIF](https://www.gbif.org/occurrence/3349116130) | 3/6/2017 | Oman | 1 | NA |
| *N. floweri* | 23.1500 | 57.2100 | [GBIF](https://www.gbif.org/occurrence/3349109631) | 3/6/2017 | Oman | 1 | NA |
| *N. floweri* | 23.4400 | 58.1000 | [GBIF](https://www.gbif.org/occurrence/3351699797) | 3/5/2017 | Oman | 1 | NA |
| *N. floweri* | 23.4600 | 58.1000 | [GBIF](https://www.gbif.org/occurrence/3351652722) | 3/5/2017 | Oman | 1 | NA |
| *N. floweri* | 23.4600 | 58.1000 | [GBIF](https://www.gbif.org/occurrence/3351633741) | 3/5/2017 | Oman | 1 | NA |
| *N. floweri* | 23.2200 | 58.0800 | [GBIF](https://www.gbif.org/occurrence/3351566923) | 3/5/2017 | Oman | 1 | NA |
| *N. floweri* | 23.4600 | 58.1000 | [GBIF](https://www.gbif.org/occurrence/3349295861) | 3/5/2017 | Oman | 1 | NA |
| *N. floweri* | 23.2200 | 58.0800 | [GBIF](https://www.gbif.org/occurrence/3349257971) | 3/5/2017 | Oman | 1 | NA |
| *N. floweri* | 23.2200 | 58.0800 | [GBIF](https://www.gbif.org/occurrence/3349256536) | 3/5/2017 | Oman | 1 | NA |
| *N. floweri* | 23.2200 | 58.0800 | [GBIF](https://www.gbif.org/occurrence/3349255924) | 3/5/2017 | Oman | 1 | NA |
| *N. floweri* | 23.4600 | 58.1000 | [GBIF](https://www.gbif.org/occurrence/3349242691) | 3/5/2017 | Oman | 1 | NA |
| *N. floweri* | 23.4600 | 58.1000 | [GBIF](https://www.gbif.org/occurrence/3349235627) | 3/5/2017 | Oman | 1 | NA |
| *N. floweri* | 23.2200 | 58.0800 | [GBIF](https://www.gbif.org/occurrence/3349204747) | 3/5/2017 | Oman | 1 | NA |
| *N. floweri* | 23.4600 | 58.1000 | [GBIF](https://www.gbif.org/occurrence/3349161633) | 3/5/2017 | Oman | 1 | NA |
| *N. floweri* | 23.4600 | 58.1000 | [GBIF](https://www.gbif.org/occurrence/3349153513) | 3/5/2017 | Oman | 1 | NA |
| *N. floweri* | 23.5800 | 57.2100 | [GBIF](https://www.gbif.org/occurrence/3349153514) | 3/2/2017 | Oman | 1 | NA |
| *N. floweri* | 23.1100 | 57.8300 | [GBIF](https://www.gbif.org/occurrence/3351630741) | 11/14/2016 | Oman | 1 | NA |
| *N. floweri* | 23.4600 | 58.1000 | [GBIF](https://www.gbif.org/occurrence/3351580657) | 11/14/2016 | Oman | 1 | NA |
| *N. floweri* | 23.1200 | 57.8400 | [GBIF](https://www.gbif.org/occurrence/3351568876) | 11/14/2016 | Oman | 1 | NA |
| *N. floweri* | 23.4400 | 58.1000 | [GBIF](https://www.gbif.org/occurrence/3349161634) | 11/14/2016 | Oman | 1 | NA |
| *N. floweri* | 23.4600 | 58.1000 | [GBIF](https://www.gbif.org/occurrence/3349072806) | 11/14/2016 | Oman | 1 | NA |
| *N. floweri* | 23.3100 | 57.4800 | [GBIF](https://www.gbif.org/occurrence/3351533885) | 11/10/2016 | Oman | 1 | NA |
| *N. floweri* | 23.3600 | 57.6400 | [GBIF](https://www.gbif.org/occurrence/3349275519) | 11/10/2016 | Oman | 1 | NA |
| *N. floweri* | 15.6095 | 32.5364 | LMZ | 20.8.1957 | Sudan | 1 | 1 female |
| *N. floweri* | 15.792392 | 38.447314 | NRM | NA | Cheren, Eritrea | 1 | NA |
| *N. floweri* | 18.204265 | -8.091414 | Roy and Leston 1975 | 31.3.1958 | Tartaft, Mauritania | 1 | 1 male |
| *N. floweri* | 17.955052 | 9.063489 | Roy and Leston 1975 | 23.8.1897 | Air oriental, Niger | 1 | 1 female |
| *N. floweri* | 16.967761 | 7.995269 | Roy and Leston 1975 | 11.1938 | Agadez, Niger | 3 | 2 males  1 female |
| *N. floweri* | 20.687043 | 18.048562 | Roy and Leston 1975 | 23.12.1958 | Tibesti Mountains, Chad | 3 | 1 male  2 females |
| *N. floweri* | 13.566713 | 33.568091 | Roy and Leston 1975 | 1906 | Sennar, Sudan | 1 | 1 male |
| *N. floweri* | 15.458535 | 32.558423 | Roy and Leston 1975 | 31.1. 1928 | Khartoum, Sudan | 1 | 1 male |
| *N. floweri* | 15.458535 | 32.558423 | Roy and Leston 1975 | 15.3.1931 | Khartoum, Sudan | 1 | 1 female |
| *N. floweri* | 13.007386 | 42.730117 | Roy and Leston 1975 | 1907 | Āssab, Eritrea | 1 | 1 male |
| *N. floweri* | 15.100546 | 36.656376 | Roy and Leston 1975 | 3.8.1935 | Teseney, Eritrea | 1 | 1 male |
| *N. floweri* | 24.566585 | 39.48105 | Roy and Leston 1975 | 16.6.1931 | Medina, Saudi Arabia | 1 | 1 male |
| *N. floweri* | 24.566585 | 39.48105 | Roy and Leston 1975 | 20.5.1938 | Medina, Saudi Arabia | 1 | 1 male |
| *N. floweri* | 21.362535 | 39.282231 | Roy and Leston 1975 | 3.10.1940 | Jeddah, Saudi Arabia | 1 | 1 male |
| *N. floweri* | 21.327369 | 39.3225 | Roy and Leston 1975 | 5.1936 | Jeddah, Saudi Arabia | 1 | 1 female |
| *N. floweri* | 21.239418 | 39.219151 | Roy and Leston 1975 | 23.11.1938 | Jeddah, Saudi Arabia | 1 | 1 male |
| *N. floweri* | 21.344608 | 39.817435 | Roy and Leston 1975 | 1931, 1932 | Mecca, Saudi Arabia | 14 | 9 males  5 females |
| *N. floweri* | 21.408093 | 40.436194 | Roy and Leston 1975 | 9.1934 | Taif, Saudi Arabia | 2 | 2 males |
| *N. floweri* | 24.619324 | 46.415803 | Roy and Leston 1975 | 9.1934 | Riyadh, Saudi Arabia | 1 | 1 female |
| *N. floweri* | 19.233845 | 41.419856 | Roy and Leston 1975 | 3.1948 | Wadi Qanuna Saudi Arabia | 1 | 1 male |
| *N. floweri* | 18.288733 | 42.780272 | Roy and Leston 1975 | 6.1936 | Khamis Mushait Saudi Arabia | 1 | 1 male |
| *N. floweri* | 17.555528 | 44.196978 | Roy and Leston 1975 | 10.1936 | Najran Saudi Arabia | 1 | 1 female |
| *N. floweri* | 21.663724 | 39.255212 | Roy and Leston 1975 | 1.1962 | Buraiman, Jeddah Saudi Arabia | 1 | 1 female |
| *N. floweri* | 12.779334 | 45.012206 | Roy and Leston 1975 | 1945, 1956 | Aden, Yemen | 7 | 6 males  1 female |
| *N. floweri* | 21.348994 | 39.171191 | Kaltenbach, 1982 | 28.9.1975 | Jeddah, Saudi Arabia | 1 | 1 female |
| *N. floweri* | 22.941914 | 41.667216 | Kaltenbach, 1982 | 24.8.1976 | Bahra, Saudi Arabia | 1 | 1 female |
| *N. floweri* | 16.968977 | 42.615031 | Kaltenbach, 1982 | 21.4.1976 | Abha-Gizan, Saudi Arabia | 1 | 1 male |
| *N. floweri* | 17.899736 | 42.380049 | Kaltenbach, 1982 | 30.09.1978 | Marabah, Saudi Arabia | 1 | 1 male |
| *N. floweri* | 17.008314 | 42.826885 | Kaltenbach, 1982 | 15.4.1979 | Hakimat Abu Arish, Saudi Arabia | 1 | 1 male |
| *N. floweri* | 16.887359 | 42.550732 | Kaltenbach, 1982 | 6.2.1980 | Jizan, Saudi Arabia | 1 | 1 male |
| *N. floweri* | 21.405725 | 39.735066 | Kaltenbach, 1982 | 9.4.1934 | Mecca, Saudi Arabia | 1 | 1 male |
| *N. floweri* | 21.383051 | 40.393225 | Kaltenbach, 1982 | 19.9.1930 | Taif, Saudi Arabia | 1 | 1 male |
| *N. floweri* | 23.719906 | 55.672807 | Kaltenbach, 1982 | 11.3.1976 | Oman | 1 | 1 female |

**Supplementary Table S2:** Information regarding the samples used in this study

| **Species** | **Latitude** | **Longitude** | **Voucher** | **Accession No.** | **Haplotype No.** |
| --- | --- | --- | --- | --- | --- |
| *Nilomantis floweri* | 27.837874 | 52.048782 | ZMPC01 | OQ223227 | H_01 |
| *Nilomantis floweri* | 27.835588 | 52.052483 | ZMPC02 | OQ223228 | H_02 |
| *Nilomantis floweri* | 27.845478 | 52.084887 | ZMPC03 | OQ223229 | H_01 |
| *Nilomantis floweri* | 27.735541 | 52.202416 | ZMPC04 | OQ223230 | H_01 |
| *Nilomantis floweri* | 27.735636 | 52.200603 | ZMPC05 | OQ223231 | H_01 |
| *Nilomantis floweri* | 27.815288 | 52.330418 | ZMPC06 | OQ223232 | H_03 |
| *Nilomantis floweri* | 27.820616 | 52.333573 | ZMPC07 | OQ223233 | H_04 |
| *Nilomantis floweri* | 27.82073 | 52.331942 | ZMPC13 | OQ223234 | H_01 |
| *Nilomantis floweri* | 27.818744 | 52.332991 | ZMPC14 | OQ223235 | H_01 |
| *Nilomantis floweri* | 27.829634 | 52.331003 | ZMPC15 | OQ223236 | H_01 |
| *Nilomantis floweri* | 26.544378 | 54.877843 | ZMPC09 | OQ223237 | H_01 |
| *Nilomantis floweri* | 26.545827 | 54.879678 | ZMPC12 | OQ223238 | H_01 |
| *Nilomantis floweri* | 27.82073 | 52.331942 | ZMCBSU01 | OQ223239 | H_01 |
| *Nilomantis floweri* | 27.829634 | 52.331003 | ZMCBSU02 | OQ223240 | H_04 |
| *Nilomantis floweri* | 27.735826 | 52.198468 | ZMCBSU03 | OQ223241 | H_01 |
| *Nilomantis floweri* | 27.735232 | 52.198509 | ZMCBSU04 | OQ223242 | H_01 |
| *Nilomantis floweri* | 27.815288 | 52.330418 | ZMCBSU05 | OQ223243 | H_04 |
| *Nilomantis floweri* | 26.544272 | 54.877011 | ZMCBSU08 | OQ223244 | H_05 |
| *Nilomantis floweri* | 60.627439 | 60.17222 | ESPC.N1 | OQ223245 | H_06 |
| *Nilomantis floweri* | 26.554115 | 54.889141 | ZMPC10 | OQ223246 | H_05 |
| *Nilomantis floweri* | 26.555113 | 54.889592 | ZMPC11 | OQ223247 | H_05 |
| *Nilomantis floweri* | 25.307856 | 60.62577 | ZMCBSU09 | OQ223248 | H_07 |
| *Nilomantis floweri* | 25.306202 | 60.627439 | ZMCBSU10 | OQ223249 | H_08 |
| *Nilomantis floweri* | 23.217 | 58.0776 | MK15.OM | MK950315 | H_09 |
| *Nilomantis floweri* | 22.9509 | 57.5373 | MK14.OM | MK950314 | H_09 |
| *Nilomantis floweri* | 22.9509 | 57.5373 | MK13.OM | MK950313 | H_10 |
| *Nilomantis floweri* | 23.217 | 58.0776 | MK12.OM | MK950312 | H_11 |
| *Nilomantis floweri* | 23.1507 | 57.2063 | MK11.OM | MK950311 | H_05 |
| *Nilomantis floweri* | 23.217 | 58.0776 | MK10.OM | MK950310 | H_12 |
| *Nilomantis floweri* | 23.4576 | 58.1026 | MK09.OM | MK950309 | H_13 |
| *Nilomantis floweri* | 23.217 | 58.0776 | MK08.OM | MK950308 | H_13 |
| *Nilomantis floweri* | 23.0529 | 57.4628 | MK07.OM | MK950307 | H_13 |
| *Nilomantis floweri* | 23.5763 | 57.2116 | MK06.OM | MK950306 | H_14 |
| *Nilomantis floweri* | 23.4581 | 58.1022 | MK05.OM | MK950305 | H_15 |
| *Nilomantis floweri* | 23.1321 | 58.3925 | MK04.OM | MK950304 | H_15 |
| *Nilomantis floweri* | 23.1508 | 57.2061 | MK03.OM | MK950303 | H_15 |
| *Nilomantis floweri* | 23.4434 | 58.097 | MK02.OM | MK950302 | H_16 |
| *Nilomantis floweri* | 23.3564 | 57.6363 | MK01.OM | MK950301 | H_17 |
| *Nilomantis floweri* | 23.4581 | 58.1022 | MK00.OM | MK950300 | H_17 |
| *Nilomantis floweri* | 23.0529 | 57.4628 | MK99.OM | MK950299 | H_18 |
| *Nilomantis floweri* | 23.4581 | 58.1022 | MK98.OM | MK950298 | H_18 |
| *Nilomantis floweri* | 23.217 | 58.0776 | MK97.OM | MK950297 | H_05 |
| *Nilomantis floweri* | 23.1507 | 57.2063 | MK96.OM | MK950296 | H_12 |
| *Nilomantis floweri* | 23.4581 | 58.1022 | MK95.OM | MK950295 | H_05 |
| *Nilomantis floweri* | 23.3066 | 57.4778 | MK94.OM | MK950294 | H_05 |
| *Nilomantis floweri* | 23.3066 | 57.4778 | MK93.OM | MK950293 | H_19 |
| *Nilomantis floweri* | 22.9571 | 57.5367 | MK92.OM | MK950292 | H_20 |
| *Nilomantis floweri* | 23.4576 | 58.1026 | MK91.OM | MK950291 | H_20 |
| *Nilomantis floweri* | 23.1216 | 57.8392 | MK90.OM | MK950290 | H_20 |
| *Nilomantis floweri* | 23.0529 | 57.4628 | MK89.OM | MK950289 | H_20 |
| *Nilomantis floweri* | 23.1062 | 57.8334 | MK88.OM | MK950288 | H_21 |
| *Nilomantis floweri* | 23.4434 | 58.0969 | MK87.OM | MK950287 | H_22 |
| *Nilomantis floweri* | 23.1507 | 57.2063 | MK86.OM | MK950286 | H_23 |
| *Nilomantis floweri* | 23.1321 | 58.3925 | MK85.OM | MK950285 | H_24 |
| *Nilomantis floweri* | 23.4581 | 58.1022 | MK84.OM | MK950284 | H_23 |
| *Nilomantis floweri* | 23.4581 | 58.1022 | MK83.OM | MK950283 | H_23 |
| *Nilomantis floweri* | 23.3066 | 57.4778 | MK82.OM | MK950282 | H_25 |
| *Nilomantis floweri* | 23.3066 | 57.4778 | MK81.OM | MK950281 | H_26 |
| *Nilomantis floweri* | 22.9571 | 57.5367 | MK80.OM | MK950280 | H_27 |
| *Nilomantis floweri* | 23.4576 | 58.1026 | MK79.OM | MK950279 | H_28 |

**Supplementary Table S3:** Performance of 84 models created during the evaluation process for *N. floweri*. The table displays the set of selected variables including set 1: the maximum temperature of the warmest month (Bio5), minimum temperature of the coldest month (Bio6), annual precipitation (Bio12), precipitation of driest quarter (bio17); set 2: containing annual mean temperature (Bio1), the maximum temperature of the warmest month (Bio5), annual precipitation (Bio12), precipitation of driest month (Bio14); and set 3: containing maximum temperature of the warmest month (Bio5), minimum temperature of the coldest month (Bio6), annual precipitation (Bio12), precipitation of driest month (Bio14).; the seven feature classes representing combinations of linear (L), quadratic (Q) and product (P); the four investigated regularisation multipliers (0.1; 0.5; 1.0; 1.5); partial ROC (partial Receiver Operating Characteristic; *p-values*); omission rate at 5 %; Akaike Information Criterion corrected (AICc); delta AIC (∆AIC; score to measure the difference between the best model (smallest AIC) and each model); Akaike Information Criterion weights (W AIC) and total number of parameters per setting. Selected parameters used in this study are shown in grey blocking.

| **Set** | **Feature classes** | **Reg. Multiplier** | **Mean AUC** | **partial ROC (*p-value*)** | **Omission rate at 5%** | **AICc** | **∆AIC** | **W AIC** | **Number of parameters** |
| --- | --- | --- | --- | --- | --- | --- | --- | --- | --- |
| 1 | l | 0.1 | 1.085 | 0 | 0.056 | 1526.398 | 5.835 | 0.001 | 4 |
| 1 | lp | 0.1 | 1.074 | 0 | 0.167 | 1523.133 | 2.569 | 0.003 | 4 |
| 1 | lq | 0.1 | 1.071 | 0 | 0.278 | 1524.589 | 4.026 | 0.002 | 6 |
| 1 | lqp | 0.1 | 1.078 | 0 | 0.167 | 1523.399 | 2.836 | 0.003 | 8 |
| 1 | p | 0.1 | 1.078 | 0 | 0.167 | 1520.563 | 0.000 | 0.014 | 8 |
| 1 | q | 0.1 | 1.091 | 0 | 0.167 | 1524.261 | 3.697 | 0.002 | 10 |
| 1 | qp | 0.1 | 1.070 | 0 | 0.167 | 1526.318 | 5.755 | 0.001 | 13 |
| 1 | l | 0.5 | 1.085 | 0 | 0.056 | 1526.399 | 5.835 | 0.001 | 4 |
| 1 | lp | 0.5 | 1.076 | 0 | 0.167 | 1523.236 | 2.672 | 0.005 | 7 |
| 1 | lq | 0.5 | 1.08 | 0 | 0.167 | 1528.674 | 8.111 | 0 | 8 |
| 1 | lqp | 0.5 | 1.088 | 0 | 0.167 | 1529.394 | 8.831 | 0 | 10 |
| 1 | p | 0.5 | 1.061 | 0 | 0.222 | 1526.682 | 6.119 | 0.001 | 6 |
| 1 | q | 0.5 | 1.066 | 0 | 0.167 | 1523.139 | 2.576 | 0.004 | 4 |
| 1 | qp | 0.5 | 1.085 | 0 | 0.167 | 1526.778 | 6.215 | 0.001 | 9 |
| 1 | l | 1 | 1.081 | 0 | 0.111 | 1526.43 | 5.866 | 0.001 | 4 |
| 1 | lp | 1 | 1.073 | 0 | 0.167 | 1523.658 | 3.095 | 0.006 | 5 |
| 1 | lq | 1 | 1.065 | 0 | 0.167 | 1526.372 | 5.808 | 0.001 | 6 |
| 1 | lqp | 1 | 1.071 | 0 | 0.167 | 1529.573 | 9.010 | 0 | 8 |
| 1 | p | 1 | 1.068 | 0 | 0.167 | 1526.26 | 5.696 | 0.001 | 4 |
| 1 | q | 1 | 1.077 | 0 | 0.167 | 1523.232 | 2.668 | 0.006 | 4 |
| 1 | qp | 1 | 1.07 | 0 | 0.167 | 1526.827 | 6.264 | 0.001 | 7 |
| 1 | l | 1.5 | 1.074 | 0 | 0.111 | 1526.494 | 5.930 | 0.002 | 4 |
| 1 | lp | 1.5 | 1.066 | 0 | 0.167 | 1523.654 | 3.090 | 0.011 | 4 |
| 1 | lq | 1.5 | 1.065 | 0 | 0.167 | 1527.715 | 7.152 | 0.001 | 6 |
| 1 | lqp | 1.5 | 1.065 | 0 | 0.167 | 1527.883 | 7.319 | 0.002 | 6 |
| 1 | p | 1.5 | 1.067 | 0 | 0.111 | 1524.436 | 3.872 | 0.006 | 3 |
| 1 | q | 1.5 | 1.074 | 0 | 0.167 | 1523.406 | 2.842 | 0.009 | 4 |
| 1 | qp | 1.5 | 1.065 | 0 | 0.167 | 1528.039 | 7.476 | 0.001 | 6 |
| 2 | l | 0.1 | 1.063 | 0 | 0.167 | 1529.211 | 8.648 | 0 | 4 |
| 2 | lp | 0.1 | 1.059 | 0 | 0.167 | 1528.015 | 7.451 | 0 | 8 |
| 2 | lq | 0.1 | 1.07 | 0 | 0.167 | 1525.085 | 4.521 | 0.001 | 8 |
| 2 | lqp | 0.1 | 1.072 | 0 | 0.167 | 1534.144 | 13.581 | 0 | 12 |
| 2 | p | 0.1 | 1.062 | 0 | 0.167 | 1535.842 | 15.278 | 0 | 6 |
| 2 | q | 0.1 | 1.066 | 0 | 0.167 | 1525.508 | 4.945 | 0.001 | 4 |
| 2 | qp | 0.1 | 1.078 | 0 | 0.167 | 1524.095 | 3.532 | 0.002 | 7 |
| 2 | l | 0.5 | 1.062 | 0 | 0.167 | 1529.224 | 8.661 | 0 | 4 |
| 2 | lp | 0.5 | 1.071 | 0 | 0.167 | 1530.13 | 9.566 | 0 | 5 |
| 2 | lq | 0.5 | 1.081 | 0 | 0.167 | 1527.786 | 7.223 | 0 | 7 |
| 2 | lqp | 0.5 | 1.076 | 0 | 0.167 | 1528.485 | 7.921 | 0 | 6 |
| 2 | p | 0.5 | 1.058 | 0 | 0.167 | 1533.874 | 13.310 | 0 | 5 |
| 2 | q | 0.5 | 1.076 | 0 | 0.167 | 1525.577 | 5.014 | 0.001 | 4 |
| 2 | qp | 0.5 | 1.077 | 0 | 0.167 | 1526.105 | 5.542 | 0.001 | 5 |
| 2 | l | 1 | 1.063 | 0 | 0.167 | 1529.295 | 8.732 | 0 | 4 |
| 2 | lp | 1 | 1.055 | 0 | 0.167 | 1528.442 | 7.878 | 0.001 | 4 |
| 2 | lq | 1 | 1.08 | 0 | 0.167 | 1529.518 | 8.955 | 0 | 6 |
| 2 | lqp | 1 | 1.063 | 0 | 0.167 | 1530.98 | 10.416 | 0 | 6 |
| 2 | p | 1 | 1.057 | 0 | 0.167 | 1530.195 | 9.632 | 0 | 3 |
| 2 | q | 1 | 1.075 | 0 | 0.167 | 1525.907 | 5.343 | 0.002 | 4 |
| 2 | qp | 1 | 1.065 | 0 | 0.167 | 1528.615 | 8.051 | 0.001 | 5 |
| 2 | l | 1.5 | 1.057 | 0 | 0.167 | 1529.427 | 8.864 | 0 | 4 |
| 2 | lp | 1.5 | 1.053 | 0 | 0.167 | 1529.14 | 8.576 | 0.001 | 4 |
| 2 | lq | 1.5 | 1.074 | 0 | 0.167 | 1528.524 | 7.961 | 0.001 | 5 |
| 2 | lqp | 1.5 | 1.064 | 0 | 0.167 | 1529.656 | 9.093 | 0.001 | 5 |
| 2 | p | 1.5 | 1.056 | 0 | 0.167 | 1530.301 | 9.738 | 0 | 3 |
| 2 | q | 1.5 | 1.065 | 0 | 0.167 | 1526.438 | 5.874 | 0.002 | 4 |
| 2 | qp | 1.5 | 1.067 | 0 | 0.167 | 1529.639 | 9.075 | 0.001 | 5 |
| 3 | l | 0.1 | 1.084 | 0 | 0.056 | 1524.831 | 4.268 | 0.001 | 4 |
| 3 | lp | 0.1 | 1.088 | 0 | 0.167 | 1526.332 | 5.768 | 0.001 | 9 |
| 3 | lq | 0.1 | 1.081 | 0 | 0.111 | 1522.505 | 1.942 | 0.005 | 8 |
| 3 | lqp | 0.1 | 1.076 | 0 | 0.167 | 1522.376 | 1.813 | 0.006 | 12 |
| 3 | p | 0.1 | 1.071 | 0 | 0.111 | 1528.158 | 7.594 | 0 | 6 |
| 3 | q | 0.1 | 1.084 | 0 | 0.167 | 1521.696 | 1.132 | 0.007 | 4 |
| 3 | qp | 0.1 | 1.085 | 0 | 0.167 | 1523.666 | 3.103 | 0.003 | 10 |
| 3 | l | 0.5 | 1.079 | 0 | 0.056 | 1524.83 | 4.267 | 0.002 | 4 |
| 3 | lp | 0.5 | 1.064 | 0 | 0.167 | 1522.249 | 1.686 | 0.008 | 6 |
| 3 | lq | 0.5 | 1.099 | 0 | 0.167 | 1527.802 | 7.238 | 0 | 8 |
| 3 | lqp | 0.5 | 1.083 | 0 | 0.167 | 1521.22 | 0.656 | 0.014 | 7 |
| 3 | p | 0.5 | 1.063 | 0 | 0.167 | 1524.661 | 4.098 | 0.002 | 4 |
| 3 | q | 0.5 | 1.081 | 0 | 0.167 | 1521.739 | 1.175 | 0.009 | 4 |
| 3 | qp | 0.5 | 1.076 | 0 | 0.167 | 1521.266 | 0.703 | 0.014 | 7 |
| 3 | l | 1 | 1.084 | 0 | 0.111 | 1524.865 | 4.302 | 0.002 | 4 |
| 3 | lp | 1 | 1.065 | 0 | 0.167 | 1525.175 | 4.611 | 0.003 | 6 |
| 3 | lq | 1 | 1.084 | 0 | 0.167 | 1524.676 | 4.113 | 0.003 | 6 |
| 3 | lqp | 1 | 1.07 | 0 | 0.167 | 1530.958 | 10.394 | 0 | 9 |
| 3 | p | 1 | 1.061 | 0 | 0.167 | 1523.977 | 3.413 | 0.004 | 3 |
| 3 | q | 1 | 1.08 | 0 | 0.167 | 1521.996 | 1.433 | 0.011 | 4 |
| 3 | qp | 1 | 1.076 | 0 | 0.167 | 1528.214 | 7.650 | 0.001 | 8 |
| 3 | l | 1.5 | 1.074 | 0 | 0.111 | 1524.938 | 4.375 | 0.004 | 4 |
| 3 | lp | 1.5 | 1.064 | 0 | 0.167 | 1525.007 | 4.443 | 0.006 | 5 |
| 3 | lq | 1.5 | 1.071 | 0 | 0.167 | 1526.011 | 5.447 | 0.003 | 6 |
| 3 | lqp | 1.5 | 1.072 | 0 | 0.167 | 1534.691 | 14.127 | 0 | 9 |
| 3 | p | 1.5 | 1.061 | 0 | 0.167 | 1524.215 | 3.651 | 0.007 | 3 |
| 3 | q | 1.5 | 1.074 | 0 | 0.167 | 1522.416 | 1.852 | 0.016 | 4 |
| 3 | qp | 1.5 | 1.074 | 0 | 0.167 | 1532.165 | 11.602 | 0 | 8 |
